# Hyaluronic Acid Decoration Facilitates CD44‐Mediated Targeting and Alters Protein Corona Formation of Extracellular Vesicles

**DOI:** 10.1002/jev2.70263

**Published:** 2026-03-26

**Authors:** Heikki Kyykallio, Elena Scurti, Martina Hanzlíková, Kirsti Härkönen, Tuovi Salo, Veera Keinonen, Janne Capra, Tapani Viitala, Jaakko Teppo, Tatu Lajunen, Kirsi Rilla

**Affiliations:** ^1^ Institute of Biomedicine, School of Medicine, Faculty of Health Sciences University of Eastern Finland Kuopio Finland; ^2^ Drug Research Program and Division of Pharmaceutical Chemistry and Technology, Faculty of Pharmacy University of Helsinki Helsinki Finland; ^3^ Department of Technical Physics, Faculty of Science Forestry and Technology University of Eastern Finland Kuopio Finland; ^4^ Pharmaceutical Sciences Laboratory, Faculty of Science and Engineering Åbo Akademi University Turku Finland; ^5^ School of Pharmacy, Faculty of Health Sciences University of Eastern Finland Kuopio Finland

**Keywords:** biomolecular corona, CD44, extracellular vesicle, glycan, hyaluronic acid, protein corona

## Abstract

Cell recognition and uptake of extracellular vesicles (EVs) is mediated by a variety of surface molecules. Growing interest has recently been drawn towards glycan structures on EVs. Hyaluronic acid (HA) is a negatively charged glycosaminoglycan that can decorate the surface of EVs from different origins. HA is a ligand for adhesion receptor CD44, which is overexpressed in various cancers and inflammatory diseases. Although HA has been utilised as a surface decoration to improve CD44‐mediated targeting of synthetic nanoparticles and EVs, the role of CD44 in the uptake of EVs is not well known. To assess the importance of CD44 in the interactions and endocytosis of HA‐decorated EVs, uptake of HA‐decorated and nondecorated EVs into CD44‐expressing and ‐deficient cells was investigated using microscopic methods. The uptake of HA‐decorated EVs was significantly increased into cells that expressed CD44, but no differences in endocytosis mechanisms were found. Additionally, the formation of plasma‐derived protein corona in HA‐decorated and nondecorated EVs was investigated using multi‐parametric surface plasmon resonance and mass spectrometry. HA‐decoration was found to cause a formation of thicker corona and enrichment of plasma‐derived protein corona components. These findings highlight the role of HA in enhancing CD44‐mediated EV targeting and modulating the composition of protein corona.

## 1 Introduction

Extracellular vesicles (EVs) harbour a rich array of biomolecules on their surface, allowing them to interact with various cell types, tissues and biological fluids. The components of the EV surfaceome include a variety of lipids, proteins, nucleic acids and polysaccharides, which can be acquired either intrinsically during EV biogenesis or from the extracellular environment (Hallal et al. 2022). The binding and uptake of EVs into cells can be facilitated by surface proteins such as integrins (Altei et al. 2020; Hoshino et al. 2015), ICAMs (Zhang et al. 2022) or by surface glycans (Christianson et al. 2013; Williams et al. 2019). Glycosaminoglycan hyaluronic acid (HA) that can decorate the surface of EVs (Härkönen et al. 2019; Paul et al. 2020; Rilla et al. 2013) has also been suggested to mediate the interactions between EVs and cells (Babula et al. 2023; Lim et al. 2021). HA has been extensively studied as a surface decoration on synthetic nanoparticles to improve their targeting and physicochemical properties (Kotla et al. 2021; Mizrahy et al. 2011).

HA is an anionic glycosaminoglycan polymer, which consist of repeating units of glucuronic acid and N‐acetylglucosamine. It is one of the main components of the extracellular matrix (ECM) and in humans synthesised and secreted to the extracellular space by three transmembrane enzyme isoforms, hyaluronan synthases 1–3 (HAS1, HAS2 and HAS3) (Weigel and DeAngelis 2007). Under normal conditions, HA is produced as a high‐molecular weight polymer (molecular mass from ∼100 kDa up to >10 MDa), but under stress or disease, it is broken down into low‐molecular weight forms (Cyphert et al. 2015). HA, along with other glycosaminoglycans and proteoglycans, forms the glycocalyx that coats different cell types (Möckl 2020), but HA has also a multifunctional role as a signalling molecule mediating cellular processes like proliferation and wound healing (Dicker et al. 2014). Active synthesis of HA has also been shown to stimulate formation of filopodia and membrane protrusions (Koistinen et al. 2015; Shurer et al. 2019), leading to shedding of HA‐decorated EVs from the tips of the formed protrusions (Arasu et al. 2020; Capra et al. 2022; Noble et al. 2020). HA‐decorated EVs are naturally secreted by various cell types with active endogenous HA‐synthesis, including mesenchymal stem cells (Arasu et al. 2017) and various cancer cells (Aaltonen et al. 2022; Babula et al. 2023; Paul et al. 2020; Pendiuk Goncalves et al. 2023).

The attachment of HA to the cell membrane is mediated by several receptors, including cluster of differentiation 44 (CD44), receptor for hyaluronic acid‐mediated motility (RHAMM), hyaluronan receptor for endocytosis (HARE), lymphatic vessel endothelial hyaluronan receptor 1 (LYVE‐1), toll‐like receptors (TLRs) and Layilin (Carvalho et al. 2023). Amongst these, CD44 – a transmembrane glycoprotein involved in cell adhesion and migration – is recognised as the principal HA‐binding receptor (Chen et al. 2018). It has been widely studied as the target for HA‐decorated nanoparticle‐based therapies (Kesharwani et al. 2022), as CD44 expression is highly upregulated in various cancers (Xu et al. 2020), and inflammatory diseases (Puré and Cuff 2001). The standard isoform (CD44s) consists of an N‐terminal extracellular ligand‐binding region, a transmembrane region and an intracellular cytoplasmic tail region. The CD44 gene has a total of 19 exons, 10 of which are present in the standard isoform. Alternative splicing generates CD44 variants (CD44v), which contain additional extracellular binding motifs for growth factors and cytokines and are typically associated with the most aggressive cancer types (Chen et al. 2018).

HA has been shown to enhance targeting and uptake of self‐assembled nanoparticles (Cadete et al. 2019; Kang et al. 2021), HA‐decorated synthetic nanoparticles (Lee et al. 2020; Mansoori‐Kermani et al. 2022; Zerrillo et al. 2022), and HA‐decorated EVs (Gong et al. 2023; Lim et al. 2021; Liu et al. 2024) into CD44‐expressing cells. However, the precise functional role of CD44 in EV binding and endocytosis remains elusive, and there is conflicting evidence that CD44 itself would mediate the endocytosis of HA‐decorated nanoparticles, or EVs. In this study, we investigated the importance of CD44s for HA‐mediated EV‐cell interactions and internalisation of EVs using a gastric cancer model comprising of both CD44‐expressing and CD44‐deficient cells. Here, we found that CD44 is essential for efficient binding of HA‐decorated EVs, suggesting that it facilitates EV targeting. However, its role in EV internalisation remains unclear.

In addition to intrinsically acquired surfaceome, EVs gain soluble biomolecules on their surface extracellularly from within tissues and from biofluids. This biomolecular corona has recently gained increasing attention since the initial demonstration of plasma‐derived protein corona formation on EV surfaces (Tóth et al. 2021). Corona formation has been mainly studied in plasma, where apolipoproteins, complement factors, fibrinogen and immunoglobulins are among the most commonly identified EV corona‐associated proteins (Dietz et al. 2023; Palviainen et al. 2020; Tóth et al. 2021). Although the mechanisms of biogenesis and function of the protein corona are poorly understood, corona formation has been shown to differ between different EV types (Liam‐Or et al. 2024; Musicò et al. 2024) and implied to affect the in vitro uptake and in vivo distribution of EVs (Dietz et al. 2023; Liam‐Or et al. 2024). However, the role of other EV‐surface components on protein corona formation has not yet been studied. Therefore, in this study we address the effect of HA‐decoration on the EV‐surface on the formation and composition of plasma‐derived protein corona. We found that HA‐decorated EVs formed a significantly thicker corona compared to nondecorated EVs and were enriched with commonly reported corona‐associated proteins. These findings demonstrate, for the first time, that surface HA‐decoration directly affects protein corona formation on EVs, potentially altering their biological identity and interactions.

## 2 Materials and Methods

### 2.1 Cell Lines and Culture Conditions

The creation of doxycycline‐inducible GFP‐hyaluronan synthase 3 expressing MCF7 breast cancer cell line has been previously described in (Siiskonen et al. 2013). MCF7‐GFP‐HAS3 cells were cultured in MEM Alpha medium (Euroclone, Pero, Italy), supplemented with 5% FBS (Gibco, Thermo Fisher Scientific, Waltham, MA, USA), 2 mM L‐glutamine (Euroclone), 50 µg/mL streptomycin sulphate and 50 U/mL penicillin (Euroclone) and maintained with 50 µg/mL Hygromycin B (Invitrogen, Thermo Fisher Scientific). Cells were passaged twice a week with a split ratio of 1:5, using 0.05% trypsin (w/v) 0.02 % EDTA (w/v) (Gibco). In cell cultures for EV collection, EV‐depleted FBS was used. FBS (Gibco) was depleted of EVs by ultracentrifugation at 189,000 × *g* for 16 h, at +4°C, followed by sterile filtration with 0.22 µm pore size syringe filter (Sartorius, Göttingen, Germany).

The generation and characterisation of CD44 and MOCK‐transfected MKN74 gastric cancer cell lines has been previously described in (Härkönen et al. 2019). Both MKN74 CD44 and MKN74 MOCK cells were cultured in RPMI‐1640 medium (Euroclone), supplemented with 10% FBS (Gibco), 2 mM L‐glutamine (Euroclone), 50 µg/mL streptomycin sulphate and 50 U/mL penicillin (Euroclone). Both cell lines were passaged twice a week with a split ratio of 1:10, using 0.05% trypsin (w/v) 0.02 % EDTA (w/v) (Gibco). During experiments with EV‐treatments, EV‐depleted FBS was used.

### 2.2 EV Isolation

For EV isolation, MCF7‐GFP‐HAS3 cells were seeded onto 15 cm cell culture dishes at a density of 2.25 × 10^6^ cells/dish and incubated for 24 h. The growth medium was then replaced with medium containing EV‐depleted FBS. Upon induction of GFP‐HAS3 expression, the medium was supplemented with 1 µg/mL doxycycline (Sigma–Aldrich, St. Louis, MO, USA). After 72 h incubation, the conditioned media was collected. To remove cell debris, the collected media was first centrifuged at 1000 × *g* for 5 min. Next, the supernatant was collected and centrifuged at 2500 × *g* for 15 min, at +4°C. The supernatant was collected and centrifuged at 10,000 × *g* for 60 min, at +4°C. Finally, the supernatant was collected and small EVs were isolated by ultracentrifugation at 110,000 × *g* for 90 min, at +4°C using Beckman Optima XE‐100 ultracentrifuge equipped with Beckman Type 50.2 Ti fixed angle rotor (Beckman Coulter, Brea, CA, USA). The resulting EV pellets were suspended in 1 X PBS (sterile filtered prior with 0.22 µm filter), and the isolated EVs were stored at −80°C. For MP‐SPR experiments with MKN74 CD44 and MKN74 MOCK cells, after the initial ultracentrifugation the pellets were suspended in 1 X PBS, and the ultracentrifugation was repeated.

### 2.3 Nanoparticle Tracking Analysis

Particle concentrations and size distributions of the isolated EV samples were analysed using ZetaView PMX‐120 nanoparticle tracking video microscope (Particle Metrix GmbH, Inning am Ammersee, Germany), with the following settings: Scatter mode with 11 positions and 1 cycle at 22°C. The camera settings were as follows: sensitivity 85.0, shutter 70 and Frame Rate 30.00. Postacquisition analysis was conducted with the following parameters: Min Brightness 20, Max Area 1000, Min Area 10, Tracelength 15, nm/Class 5 and Classes/Decade 64.

The zeta potential of EVs was measured using ZetaView PMX‐120 nanoparticle tracking video microscope (Particle Metrix). The measurements were made in water with the following parameters: Stationary 2 Cycles, SL positions and ZP/Class 2.0 mV.

### 2.4 Transmission Electron Microscopy

All steps were performed in room temperature. A 10 µL drop of EV isolates were loaded on carbon coated and glow discharged nickel grids and incubated for 50 min in a humidity chamber, after which the grids were washed three times with 0.1 M phosphate buffer, pH 7.4 and fixed with 2 % paraformaldehyde in phosphate buffer for 10 min. The grids were then washed twice in phosphate buffer and five times in deionised water and contrasted using 2% neutral uranylacetate for 15 min in the dark. The grids were rinsed with deionised water and embedded using 2% methyl cellulose (25Ctp)/0.4% uranyl acetate and incubated 10 min in the dark. The methyl cellulose/uranyl acetate solution was reduced to a thin film, and the grids were dried. The grids were imaged using JEM‐2100F transmission electron microscope (JEOL Ltd, Tokyo, Japan), operated at 200 kV.

### 2.5 Western Blot

Protein extraction was done using RIPA lysis buffer (150 mM sodium chloride, 50 mM Tris, 1% Nonidet, 0.1% SDS and 0.5% sodium deoxycholate in PBS, pH 7.5), supplemented with 1 mmol/L sodium orthovanadate, 0.1 mg/mL phenylmethylsulphonyl fluoride and 300 U/mL aprotinin. The proteins were resolved using SDS‐PAGE, followed by transfer to Amersham Protran nitrocellulose membrane (GE Healthcare, Chicago, IL, USA) with 2 mA/cm^2^ current in Fastblot B43 semidry blotter (Biometra, Göttingen, Germany). Membranes were incubated with primary antibodies (Table 1) at +4°C for o/n followed by incubation with Goat anti‐Rabbit HRP secondary antibody (G21234, Invitrogen) 1:10,000 in 0.1% tween‐TBS for 1 h in RT. Pierce ECL Western Blotting substrate (Thermo Scientific) was used for HRP‐conjugated antibody detection. The blots were imaged using ChemiDoc MP Imaging System (Bio‐Rad Laboratories, Hercules, CA, USA).

**TABLE 1:** Primary antibodies used in Western blot.

Primary antibody	Dilution	Reference	Manufacturer
Anti‐actin	1:1000 in 5% BSA‐TBS	A2066	Sigma–Aldrich
Anti‐ALIX	1:1000 in 5% BSA‐TBS	92880S	Cell Signaling Technology, Danvers, MA, USA
Anti‐calnexin	1:1000 in 5% BSA‐TBS	2433S	Cell Signaling Technology
Anti‐CD63	1:500 in 5% BSA‐TBS	52090S	Cell Signaling Technology
Anti‐CD9	1:500 in 5% BSA‐TBS	13403S	Cell Signaling Technology
Anti‐TSG101	1:1000 in 5% BSA‐TBS	ab125011	Abcam, Cambridge, UK

### 2.6 ELISA‐Like Hyaluronic Acid Assay

The HA concentration from cell culture mediums and EVs was measured using ELISA‐like sandwich assay. The HA‐specific probe HABC (Wang et al. 1996) and biotinylated probe bHABC were prepared as described previously. For hyaluronan assay, Maxisorp U‐bottom 96‐well plates (Thermo Fischer Scientific) were coated with 1 µg/mL HABC in 50 mM sodium carbonate buffer (pH 9.5) for 2 h at +37°C. The plates were washed with 0.5% Tween in PBS and blocked with 1% BSA in PBS for 1 h at +37°C. Plates were then washed with 0.5% Tween in PBS and stored in −20°C overnight. Standard HA (2.5–50 ng/mL) and medium samples or EV isolates were diluted with 1% BSA in PBS were applied to the plate and incubated for 1 h at +37°C. The plates were washed with 0.5% Tween‐PBS and incubated with 1 µg/mL bHABC in 1% BSA‐PBS for 1 h, at +37°C and washed. Horseradish peroxidase‐streptavidin (#SA‐5004, Vector Laboratories, Burlingame, CA, USA) was diluted 1:20,000 with PBS and added to the wells, incubated at +37°C for 1 h and washed with 0.5% Tween‐PBS. For colour reaction, 0.5% 3,3′,5,5′‐tetramethylbenzidine in DMSO was diluted into 0.02% solution with substrate buffer [0.1 sodium‐acetate (trihydrate), 1.5 mM citric acid (monohydrate), 0.005% H_2_O_2_, pH 6.0]. The substrate solution was added to the plate, and the plate was incubated RT in the dark until sufficient colour developed (∼12 min). The reaction was stopped with 2 M H_2_SO_4_ and the absorbances were read at 450 nm using iEMS Reader MF plate reader (Labsystems, Helsinki, Finland). The concentration of HA was calculated using a linear standard curve.

### 2.7 Confocal Imaging of MCF7‐GFP‐HAS3 EVs

For EV imaging, Ibitreat 8‐well chamber glass (Ibidi GmbH) was coated with 10 µg/mL poly‐D‐lysine (Sigma–Aldrich) for o/n at +37°C. The poly‐D‐lysine solution was removed, and the wells were washed with 1 X PBS. EV isolates were added to the wells and incubated at +37°C for 3 h. To stain the EV‐associated HA, 0.25 µg/mL Alexa fluor 568‐conjugated HABC was added. The cells and EVs were imaged with Zeiss Axio Observer microscope (Carl Zeiss Microscopy GmbH, Jena, Germany), equipped with LSM800 confocal module (Carl Zeiss Microscopy GmbH) and 63 x NA 1.4 oil objective (Carl Zeiss Microscopy GmbH) using Zen Blue 2.3 software.

### 2.8 GFP‐HAS3 EV Uptake and Confocal Microscopy

MKN74 CD44, MKN74 MOCK or their cocultures (1:1) were prepared by seeding 20,000 cells per well on an IbiTreat 8‐well chamber glass (Ibidi). After 24 h incubation, the culture medium was replaced with EV‐depleted medium, and cells were treated with 1 × 10^10^ GFP‐HAS3‐EVs per well and incubated for 6 h before fixation. To block the HAS3‐EV interactions with CD44, cells were pretreated with 0.1 mg/mL 2MDa HA (Lifecore Biomedical, Chaska, MN, USA) for 1 h before adding the EVs.

The cells were fixed with 4% paraformaldehyde and blocked with 1% BSA in PBS for 20 min. To visualise CD44, anti‐CD44 antibody (#NBPI‐31488, Novus Biologicals, Bio‐Techne Ltd., Abingdon, UK) diluted 1:250 in 1% BSA‐PBS was added and incubated at +4°C for o/n. The wells were washed with phosphate buffer, and 1:2000 Alexa fluor 594 anti‐rabbit secondary antibody (#G21234, Invitrogen) was added and incubated for 1 h at RT. The nuclei were stained with 2 µg/mL DAPI for 5 min at RT. The cells were imaged with Zeiss Axio Observer microscope (Carl Zeiss Microscopy GmbH, Germany), equipped with LSM800 confocal module (Carl Zeiss Microscopy GmbH) and 63 x NA 1.4 oil objective (Carl Zeiss Microscopy GmbH) using Zen Blue 2.3 software.

The analysis of GFP‐HAS3‐EV uptake was performed using FIJI software v. 2.14.0/1.54f (Schindelin et al. 2012). The area of CD44‐positive and CD44‐negative cells was determined from maximum intensity projections of the CD44 channel. Subtract background (rolling ball radius = 50.0 pixels) and Gaussian blur [Sigma (radius) = 0.5] were used before manual thresholding, and the thresholded areas were saved as regions of interest (ROI). Nuclei from the CD44‐positive and CD44‐negative areas were counted to quantify the number of cells. To quantify the number of GFP‐positive spots in cells, a standard deviation intensity projection from GFP channel was created, followed by the subsequent steps: subtract background (rolling ball radius = 50.0 pixels), Gaussian blur [Sigma (radius) = 0.5], threshold (default) = 46, analyse particles (size = 0–20 Pixel units). GFP‐positive spots per cell were counted using the ROIs and the number of nuclei for CD44‐positive and CD44‐negative cells.

### 2.9 Correlative Light and Electron Microscopy

Gridded No. 1.5 glass‐bottom 35 mm dishes (MatTek Life Sciences, Ashland, MA, USA) were coated with 0.6 mg/mL Cultrex rat tail Collagen I (R&D Systems Inc, Minneapolis, MN, USA) in PBS for 1 h at RT. MKN74 CD44 and MKN74 MOCK cells were cocultured at a 1:1 ratio, with a total of 100,000 cells seeded per dish, and incubated for 24 h. The cells were then treated with 4 × 10^10^ GFP‐HAS3‐EVs in EV‐depleted medium and incubated for 6 h before fixation with 2% glutaraldehyde for 15 min at RT, followed by 45 min incubation at +4°C. To visualise CD44, anti‐CD44 antibody (#NBPI‐31488, Novus Biologicals) was diluted 1:250 in 1% BSA‐PBS, added to the cells and incubated at +4°C for o/n. The cells were then washed with phosphate buffer, and 1:2000 Alexa fluor 594 anti‐rabbit secondary antibody (#G21234, Invitrogen) was added and incubated for 1 h at RT. The nuclei were stained with 2 µg/mL DAPI for 5 min at RT.

The cultures were first imaged with Zeiss Axio Observer microscope, equipped with LSM800 confocal module and 63 x NA 1.4 oil objective (Carl Zeiss Microscopy GmbH) using Zen Blue 2.3 software. The cultures were then processed for scanning electron microscopy. The cells were routinely dehydrated in ascending series of ethanol and hexamethyldisilazane and coated with a thin layer of gold. For scanning electron microscopy, the cells were imaged with Zeiss Sigma HD|VP scanning electron microscope (Carl Zeiss Microscopy GmbH) operated at 5 kV. The cells from confocal microscopy images were reacquired by utilising the labelled grids of the cell culture dishes. The confocal microscopy images were then overlaid with the corresponding scanning electron microscopy images.

### 2.10 Preparation of EV‐Depleted Human Plasma

Citrate‐phosphate‐dextrose (CPD)‐anticoagulated human plasma that had been frozen within 24 h was bought from the Finnish Red Cross Blood Service (Helsinki, Finland). The blood product is for research use only, anonymised, and constitutes of nonidentifiable human material collected according to the Declaration of Helsinki. The plasma was stored at −80°C.

To isolate EVs, plasma was first centrifuged at 2500 × *g*, for 15 min at RT, and the supernatant was collected, followed by centrifugation at 5000 × *g* for 15 min at RT. The supernatant was then diluted 1:1 with 0.22 µm filtered PBS, and EVs were pelleted by ultracentrifugation at 100,000 × *g* for 2 h at +4°C using Beckman Optima XE‐100 ultracentrifuge equipped with Beckman Type 50.4 Ti fixed angle rotor (Beckman Coulter). The supernatant was collected and stored at −80°C before being used in the protein corona experiments.

### 2.11 High‐Content Imaging of EV Uptake in Live Cells

MCF7 EVs or GFP‐HAS3‐EVs were labelled with PKH26 dye kit (Sigma–Aldrich). EV‐isolates or PBS control were diluted to 1 mL with Diluent C, and 5 µL of PKH26 dye was added followed by incubation for 20 min in RT. The EV+dye solution was moved to Amicon Ultra centrifugal filter with 10 kDa molecular weight cutoff (Sigma–Aldrich), and 3 mL of 1X PBS (filtered through 0.22 µm filter) was added. The EV + dye solution was centrifuged at 7500 × *g* for 20 min at +4°C with a fixed angle rotor. To wash the labelled EVs, 1X PBS was added up to 4 mL to the concentrate and the centrifugation was repeated. The concentrate containing the labelled EVs was collected and stored at +4°C for overnight before use.

For live cell imaging of EV uptake, a 1:1 coculture of MKN74 CD44 and MKN74 MOCK cells was made by seeding a total of 15,000 cells per well on PhenoPlate 96‐well microplate (Revvity, Waltham, MA, USA). After 24 h incubation, PKH26‐labelled EVs (2 × 10^9^ or 6 × 10^9^ particles/mL) were added and incubated for 1 h before the start of imaging. Also, before imaging, CD44‐positive cells were stained with 1:200 Alexa fluor 647 anti‐human CD44 (#397512, Biolegend) and nuclei with NucBlue Fixed Cell ReadyProbes Reagent (Invitrogen), following the manufacturer's instructions. To study the endocytosis mechanisms of MCF7‐EVs and HAS3‐EVs, the cultures were pretreated with 0.1 mg/mL 2 MDa HA (Lifecore Biomedical), or endocytosis inhibitors 5 mM methyl‐β‐cyclodextrin (MβC) (Sigma–Aldrich), 25 µM 5‐(*N*‐ethyl‐*N*‐isopropyl)amiloride (EIPA) (Sigma–Aldrich), or 10 µM chlorpromazine (Sigma–Aldrich) for 1 h before adding of 6 × 10^9^/mL PKH26‐labelled EVs. The cultures were imaged with Opera Phenix Plus high‐content imaging system, equipped with automated 40 x NA 1.1 water immersion objective (Revvity), at 1 h intervals for 5 h and at 24 h timepoint, with 10–15 Z‐stacks per timepoint and per treatment imaged in one experiment.

To investigate the impact of serum or plasma‐derived corona on EV uptake, the culture medium of MCF7‐GFP‐HAS3 cells was replaced either with serum‐free medium or EV‐depleted FBS‐containing medium, and the EVs were isolated and labelled as previously. For uptake assays in MKN74 cocultures, the medium was replaced either with serum‐free medium or EV‐depleted FBS‐containing medium before the addition of EVs. To re‐establish the protein corona of EVs, labelled EVs isolated from serum‐free cultures were incubated with 5% EV‐depleted FBS (Gibco) or 5% EV‐depleted human plasma at +4°C for overnight before addition to cells. For uptake experiments, 6 × 10^9^/mL EVs were added per well, and the cultures were imaged after 5 h incubation as described above.

Image analysis was performed using Harmony 5.2 software (Revvity). Volume of PKH26‐positive particles taken up by the cells in relation to the volume of the cells was calculated to determine the amount of PKH26‐labelled EV uptake. All analyses were performed from 3D projections. Individual cells were first detected based on nuclear staining, and the volume of the cells was determined and segmented based on the cytosolic GFP‐signal. The CD44^+^ cells were separated based on the fluorescent antibody labelling. Three‐dimensional PKH26‐positive objects were segmented, and the total volume of the PKH26‐objects was normalised to the volume of cells, separately for each individual cell. The full Harmony analysis sequence used is included as Supporting Information File S2.

### 2.12 EV‐Cell Interactions Using Multi‐Parametric Surface Plasmon Resonance (MP‐SPR)

Before being used in experiments, gold‐coated sensors (Bionavis Ltd., Tampere, Finland) were cleaned by boiling them in 1:1:5 v/v solution of 30% ammonia hydroxide (Sigma–Aldrich), 30% hydrogen peroxide (Sigma–Aldrich) and Milli‐Q water for 10 min, followed by a rinse with Milli‐Q water and 70% EtOH. The cleaned sensors were stored in 70% EtOH.

To enable the adhesion of cells on the gold surface, the sensors were coated with 25 µg/mL (5 µg/cm^2^) human plasma fibronectin (Roche) diluted in PBS by incubating 1 h at RT. After the coating, MKN74 CD44 or MKN74 MOCK cells were seeded on the sensors with a cell density of 1 × 10^5^ cells/cm^2^ and incubated at +37°C, 5% CO_2_ for 48 h to obtain a 100% confluent monolayer of cells.

The MP‐SPR experiments were performed using an MP‐SPR Navi 200 Otso instrument (Bionavis Ltd.) with an angular scan range 40°–78°, two flow channels, and wavelengths of 670 and 785 nm, equipped with a manually operated Ismatec Reglo Digital peristaltic pump (7801814) and Tygon tubing with an inner diameter of 0.19 mm. The flow channel was custom made with dimensions of 1.37 mm × 7.21 mm, and volume of 5.31 µL. During experiments, RPMI‐1640 supplemented with 25 mM HEPES was used as a running buffer. All experiments were performed at + 37°C with a constant flow rate of 15 µL/min. Before starting the measurement, the sensors with confluent cell monolayers were washed twice with DPBS. After baseline stabilisation with the running buffer (∼30–40 min, signal change of max 2 mdeg/min), EV‐sample (diluted to 1.5 × 10^10^ particles/mL in PBS) and PBS control were simultaneously injected continuously for 20 min, followed by 30 min flushing with the running buffer. The SPR measurements were performed using a laser wavelength of 670 nm. The SPR reflectance spectrum was recorded between 58° and 77° with a scanning time of approximately 2.0 s.

Data were exported in ASCII format with MP‐SPR Navi Data Viewer 6.7.0.1 software (BioNavis), and SPR peak angular positions were determined using the weighted centroid method. The time of sample injection was selected as the zero point for both time and response. Signal correction was performed by subtracting the response of the PBS control channel from the sample channel to eliminate nonspecific or bulk effects.

The SPR sensors were imaged with an optical microscope before and after the measurements to evaluate the confluency and attachment of the cells to the sensors.

### 2.13 EV‐Capture and Plasma‐Derived Protein Corona Formation With MP‐SPR

For EV capture and regeneration, Regenerable avidin kit (Bionavis Ltd.) was utilised in a 220A NAALI MP‐SPR instrument (Bionavis Ltd.). A biotinylated sensor was inserted in the instrument, and the fluidic system was primed with 1X PBS (Cytiva, Nalborough, MA, USA), which was also used as the running buffer during measurements. After stabilisation of the MP‐SPR signal for 10 min, the activation of the sensor was carried on for 5 min with the activation reagent R1; successively, regenerable avidin was injected for 7 min and the excess avidin was flushed away with running buffer for 10 min. Biotinylated anti‐human CD9 antibody (#312112, Biolegend), at a concentration of 40 µg/mL, was then injected for 10 min and flushed with running buffer for another 10 min to guarantee an appropriate coverage of the sensor.

For the EV capture experiments, EVs were injected at a concentration of 7.5 × 10^10^ particles/mL for 10 min, and the excess sample was flushed with running buffer for 10 min. To detach the antibody‐captured EVs, the sensor was regenerated with the regeneration reagent R2 for 5 min while simultaneously collecting the detached EVs in Eppendorf tubes, which were then stored at –20°C for further analysis with mass spectrometry. All the injections were carried out at a flow rate of 10 µL/min.

For the protein corona formation, after the capture of EVs, an additional injection of EV‐depleted human plasma (1:1 diluted with running buffer) was carried out for 15 min. Proteins that were not tightly adhered on the surface of the EVs were removed by flushing with running buffer for 15 min.

### 2.14 MP‐SPR Data Analysis

The dual wavelength method described from Rupert et al. (2016) was used to estimate the size of the EVs. In summary, this method is based on the wavelength‐dependent nature of several SPR parameters, including the sensitivity factor (*S*), the refractive index increment (*dn*/dC*), the evanescent field decay length (*δ*) and the dimensionless correction factor (*φ*), which accounts for cases where the film thickness is comparable to or exceeds *δ*. By measuring the SPR response (*R*) at two distinct wavelengths (*λ*_1_ and *λ*_2_) and applying the known dependencies of *φ* on film thickness (or particle diameter), the thickness (*d*) can be extracted from the ratio $R_{\lambda_{1}}$/$R_{\lambda_{2}}$. The correlation among all these parameters is illustrated in Equation (1).

(1) $$\frac{R_{\lambda_{1}}}{R_{\lambda_{2}}}=\frac{S_{\lambda_{1}}}{S_{\lambda_{2}}}\;\frac{{\left(dn/dC\right)}_{\lambda_{1}}}{{\left(dn/dC\right)}_{\lambda_{2}}}\frac{\left(1-e^{-\frac{d}{\delta_{\lambda_{1}}}}\right)}{\left(1-e^{-\frac{d}{\delta_{\lambda_{2}}}}\right)}$$

LayerSolver from Bionavis Ltd., version 1.4.1, was utilised to identify the protein corona thickness and refractive index at different wavelengths, as illustrated by Kari et al. (2017). The layer thickness (*d*) and refractive index (*n*) were modelled and calculated using SPR Navi LayerSolver v. 1.4.1 (BioNavis Ltd., Finland). The analysis was based on the two‐wavelength method, in which the fitted full SPR spectra obtained simultaneously with 670 and 785 nm lasers at the same time point were compared with the experimental spectra obtained from the measurements. LayerSolver enables the processing of multiple SPR spectra and applies a stepwise calculation approach, where the biophysical properties of molecular layers are resolved sequentially through numerical iteration, one layer at a time, considering that the deposition of the material is uniformly distributed in a homogenous layer. The embedded algorithms iteratively solve thickness and refractive index values simultaneously. For the modelling, the following input parameters were applied: for MCF7 and HAS3‐EVs, a thickness range of 80–120 nm with refractive index *n* = 1.334–1.335; and for the corona layer, a thickness range of 1–10 nm with *n* = 1.36–1.37. In the optical model, thickness values were defined as global variables for both wavelengths and fixed when calculating the successive layer thickness, while refractive indices were treated as independent variables during the iteration cycles. The starting parameters to fit the experimental spectra are described in Supporting Information (Table S1).

### 2.15 NanoLC‐MS Analysis of the EV Protein Corona

The eluates from the SPR experiments containing EVs and the protein corona were collected, processed and analysed with nanoflow liquid chromatography‐mass spectrometry (nLC‐MS).

For protein extraction, 100 µL of sample was adjusted to a pH of 8 using 1 M NaOH (Sigma–Aldrich, St. Louis, USA), and 75 mg of urea (Sigma–Aldrich) was added to the samples. Then, the samples were vortexed and sonicated for 15 min and finally centrifuged for another 15 min at 14,800 × *g*. After these procedures, the supernatants were collected and processed for protein reduction and carbamidomethylation: dithiothreitol (DTT) (Sigma–Aldrich) was firstly added to the samples to the final concentration of 10 mM, followed by incubation at room temperature for 1 h. Secondly, iodoacetamide (IAA) (Sigma–Aldrich) was added to the final concentration of 50 mM and further incubated at room temperature for 1 h. Eventually, to quench the carbamidomethylation reaction, additional DTT at the final concentration of 20 mM was added to the samples before protein precipitation.

In order to remove the detergent residues after collecting EVs from SPR, as well as excess DTT and IAA, protein precipitation was carried out with chloroform/methanol/water (4 volumes/1 volume/3 volumes) extraction to let the protein layer deposit at the interface between the aqueous and the organic solvents. At the end of the procedure, the aqueous phase was removed, and 1 volume of methanol was further added. The samples were then mixed and centrifuged for 10 min at 14,800 × *g* to pellet the proteins. Successively, the pellets were resolubilised with 25 µL of a solution of 8 M urea and 20 mM EPPS, vortexed and sonicated for 15 min.

The total protein content of the samples was determined using Pierce BCA Protein Assay Kit (ThermoFischer, Rockford, IL, USA), and thereafter the samples were diluted with 20 mM EPPS to get 1.5 M urea concentration. To normalise the samples based on total protein content, 12 µg of protein was taken from all samples for subsequent steps. Sequencing grade modified trypsin (PROMEGA, Madison, WI, USA) was dissolved in 20 mM EPPS at pH ∼8 and added to proteins in a 1:50 enzyme‐to‐protein ratio and incubated overnight at 37°C with shaking.

Protein labelling was performed by adding tandem mass tags (TMTs) to the peptide solutions: TMT16plex Kit dry reagents (ThermoFischer, Germany) were dissolved in 80 µL of anhydrous acetonitrile (ACN), and then the solutions were added to each sample in order to get 20% v/v as final ACN concentration of and a 5:1 TMT:protein ratio. The samples were successively incubated for 1 h at room temperature, followed by quenching the labelling reaction by adding 1 µL of 5% hydroxylamine (Sigma–Aldrich). Eventually, the samples were incubated for 15 min at room temperature, combined into one Eppendorf tube and dried for at least 2 h in a vacuum centrifuge to evaporate most of the acetonitrile in them.

The samples were acidified to pH ∼2 with 10% formic acid, followed by purification using C18 solid phase extraction (SPE) centrifuge cartridges (BioPureSPN Mini Proto 300 C18, 7–70 µg capacity, The Nest Group, Inc.). The SPE cartridges were conditioned with 2 × 200 µL of acetonitrile and equilibrated with 2 × 200 µL of 2% acetonitrile with 0.1% formic acid. The samples were loaded onto the cartridges and washed with 200 µL of 2% acetonitrile with 0.1% formic acid. Elution was performed first with 200 µL of 50% acetonitrile with 0.1% formic acid, followed by 200 µL of 80% acetonitrile with 0.1% formic acid. The eluted samples were evaporated to dryness using a vacuum centrifuge.

To increase the proteome coverage of the analysis, the samples were fractionated offline with high‐pH reversed‐phase liquid chromatography. The multiplexed samples were dissolved in 20 mM ammonium hydroxide (11 µL), used also as Eluent A and separated into 96 fractions on an XBridge BEH C18 2.1 × 150 mm column using a Waters Acquity UPLC system over a 48‐min gradient from 1% to 63% B (B = 20 mM ammonium hydroxide in acetonitrile) in three steps (1%–26% B in 42 min, 26%–54% B in 4 min and then 54%–63% B in 2 min) at a flow rate of 200 µL/min. Ninety‐six initial fractions were collected onto a 96‐well plate and then concatenated column‐wise into 12 final fractions. Finally, the samples were evaporated to dryness using a vacuum centrifuge.

Before LC‐MS analysis, the samples were reconstituted in 19.6 µL of Eluent A (2% acetonitrile and 0.1% formic acid in water) by 15‐min sonication to get a peptide concentration of 0.5 µg/µL and transferred into LC autosampler vials. LC‐MS analysis was conducted with an EASY‐nLC 1200 coupled to an Orbitrap Fusion mass spectrometer. Three microliters of the peptide solution were injected onto the C18 column system, consisting of a trap column (Thermo Acclaim PepMap 100, dimensions 75 µm × 2 cm, particle size 3 µm) and an analytical column (Thermo Acclaim PepMap RSLC, dimensions 75 µm × 15 cm, particle size 2 µm). The flow rate was 250 nL/min, and the gradient was as follows: 5 min with 3% Eluent B, from 3% to 19% Eluent B in 73 min, from 19% to 29% Eluent B in 28 min, from 29% to 41% Eluent B in 20 min and from 41% to 99% Eluent B in 3 min. Then, the column was washed for 7 min with 99% Eluent B, followed by decreasing the Eluent B percentage to 3% in 1 min, and 13 min flush at 3% Eluent B. Eluent A was 2% acetonitrile and 0.1% formic acid in water, and Eluent B was 90% acetonitrile and 0.1% formic acid in water. The peptides were ionised in positive ionisation mode with an electrospray voltage of 2000 V. Data‐dependent acquisition with a cycle time of 3 s was used, in which precursors from each orbitrap MS1 full scan were fragmented and analysed with MS2 in the orbitrap. EASY‐IC internal mass calibration with fluoranthene was used at the start of the run. MS1 parameters were as follows: resolution 120,000, scan range 375–1500 *m*/*z*, maximum injection time 50 ms and normalised AGC target 100%. MS2 parameters were as follows: quadrupole isolation window 1 *m*/*z*, HCD normalised collision energy 35%, resolution 50,000, automatic scan range starting from 110 *m*/*z*, maximum injection time 150 ms, normalised AGC target 200%. Precursors were limited to peptides with monoisotopic precursor selection, charge states from 2 to 7, and intensities above 10,000. Precursor fit with 50% fit error and 1 *m/z* window was applied. A 60‐s dynamic exclusion with 10 ppm mass tolerance was applied.

### 2.16 NanoLC‐MS Data Analysis

Protein identification and quantification was performed with MaxQuant, version 2.6.7.0., through Andromeda search engine (Cox and Mann 2012). The parameters used for the peptide search, against Uniprot human (20,417 proteins downloaded on 15 January 2025), (The UniProt Consortium 2025) were: unique and razor peptide search with methionine oxidation and protein N‐terminal acetylation as variable modifications and cysteine carbamidomethylation as fixed modification and the digestion type is set to “Trypsin/P”; the maximum peptide length is seven amino acids and the maximum peptide mass is 4600 Da; contaminants are included in the search and the decoy type used is “revert” decoys; the quantification type is based on MS2 reporter and the isobaric label used is the TMTpro 16‐plex.

Further data analysis was conducted with Perseus v2.1.3. (Tyanova and Cox 2018). Potential contaminant proteins and proteins with fewer than three valid values in all sample groups were removed from the results. The intensities were normalised, dividing them by the summed intensity of the sample group and multiplied by the maximum of the summed intensities across all samples. Finally, for the imputation of missing values, they were replaced by random numbers that are drawn from a normal distribution in each protein group. The parameters of the distribution are set in a way to simulate a typical abundance region that the missing values would have if they were measured: the parameters set in Perseus for the imputation were: with 0.3 and downshift 1.8.

For statistical analysis, the protein abundances between two different sample groups were compared with *t* tests, and the calculated values were multiple hypothesis corrected according to the Benjamini–Hochberg correction. Heatmap for plasma‐exposed EV protein content visualisation was generated from log_2_‐transformed intensity values using pheatmap package in R version 4.4.2. Functional enrichment analyses were performed using DAVID 2021 (Sherman et al. 2022) with DAVID knowledgebase v2023q4 and visualised using R version 4.4.2.

### 2.17 Statistics

Statistical tests for GFP‐HAS3 EV characterisation and uptake experiments were performed using GraphPad Prism version 5.03.

For high content screening uptake analyses, statistics were performed using linear mixed models in R (packages lme4, lmerTest, emmeans), version 4.4.2. Time‐based uptake was log transformed and modelled as: uptake_log ∼ cell_line * time + (1 | well). Type III ANOVA with Satterthwaite degrees of freedom was used to test for fixed effects. Estimated marginal means were obtained with emmeans. CD44+ versus CD44− comparisons were performed within each time point with Holm adjustment. Endocytosis inhibitor experiments were modelled as: uptake ∼ cell_type * treatment + (1 | experiment/well), with type III ANOVA with Satterthwaite degrees of freedom used to test for fixed effects. Tukey‐adjusted pairwise comparisons were performed for (a) cell‐type differences within each treatment and (b) treatment effects within each cell type. Corona‐associated uptake experiments were modelled as: uptake_log ∼ cell_line * condition + (1 | experiment) + (1 | well). Type III ANOVA tested fixed effects. Estimated marginal means and Tukey‐adjusted pairwise comparisons were obtained with emmeans. For within‐pair differences, the log ratio (CD44+/CD44−) was modelled as: log_ratio_A_over_B ∼ condition + (1 | experiment).

## 3 Results

### 3.1 HA‐Decorated GFP‐HAS3‐EVs Share Typical Characteristics With Nondecorated EVs

**FIGURE 1:**
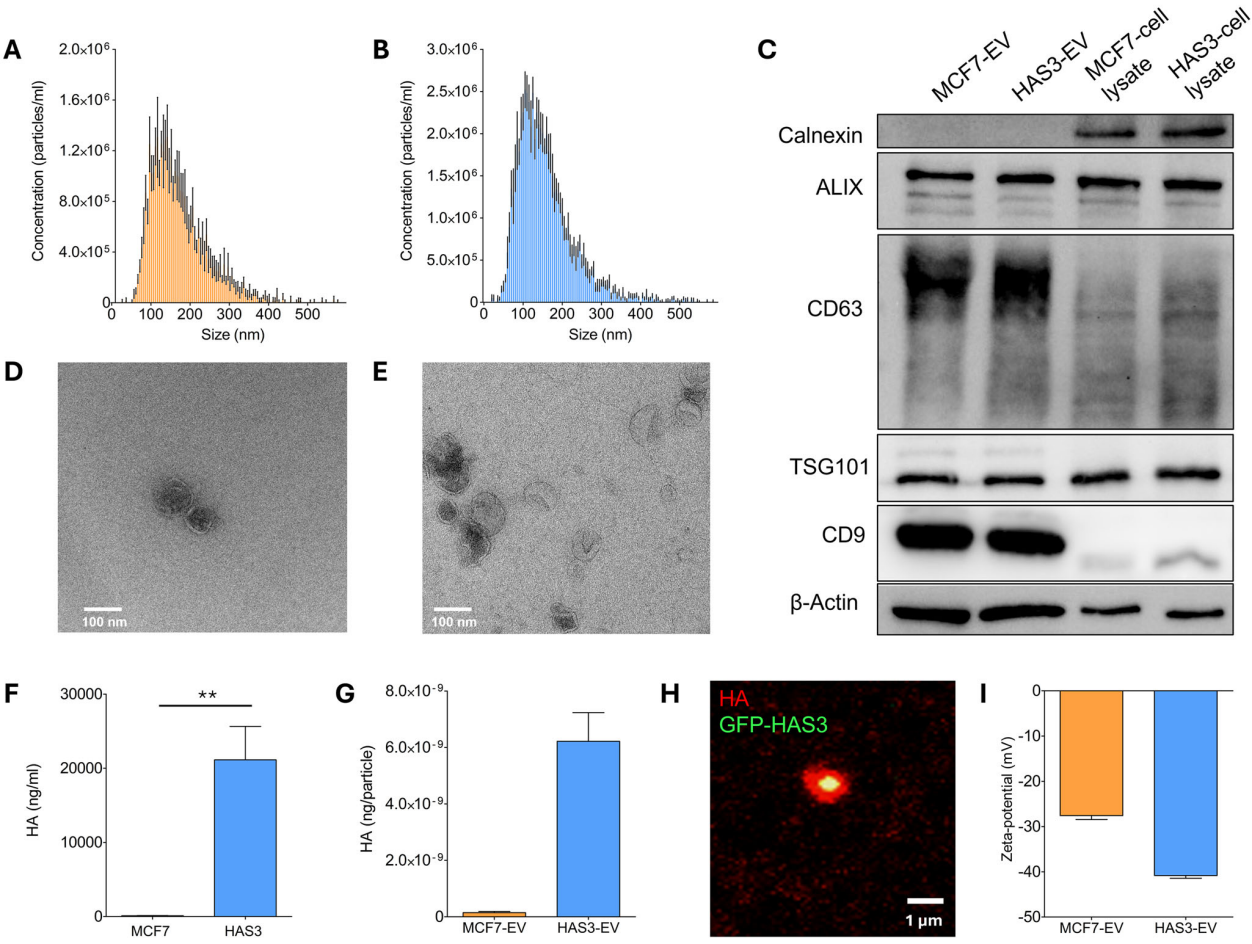
Characterisation of MCF7‐EVs and HAS3‐EVs. Nanoparticle tracking analysis of MCF7‐EVs (A) and HAS3‐EVs (B) showed a similar size distribution for the EVs (*n* = 5, mean with SEM). (C) Western blot of cytosolic EV markers ALIX and TSG101, membrane tetraspanins CD63 and CD9, and negative marker Calnexin from the EV‐isolates. Transmission electron microscopy images of MCF7‐EVs (D) and HAS3‐EVs (E) showing the morphology of isolated EVs. Concentration of HA measured from cell culture medium supernatants from MCF7 and MCF7‐HAS3 cells (F) (*n* = 6, Mann–Whitney *U* test, **p < 0.01) and amount of HA per particle in EV isolates (G) (*n* = 2–3, SEM). (H) Representative confocal microscopy image of HA‐decorated HAS3‐EV. (I) Zeta potentials of MCF7‐EVs and HAS3‐EVs (*n* = 3, SEM). EV, extracellular vesicle; HA, hyaluronic acid.

MCF7 breast epithelial cancer cell line with doxycycline‐inducible expression of GFP‐HAS3 was used to produce HA‐decorated EVs (Rilla et al. 2013; Siiskonen et al. 2013). In their native state, MCF7 cells produced low concentrations of HA, but expression of HAS3 massively increased HA synthesis and induced shedding of HA‐decorated GFP‐HAS3‐positive EVs from the tips of formed filopodia‐like protrusions (Figure S1). Consistent with previous studies (Rilla et al. 2013), expression of HAS3 increased HA secretion by nearly 200‐fold compared to noninduced MCF7 cells (Figure 1F). After isolation, EVs from non‐HAS3‐induced cells (MCF7‐EV) contained barely detectable amounts of HA, while EVs from GFP‐HAS3‐induced cells (HAS3‐EV) carried a HA coat (Figure 1G, H, Figure S1). Zeta‐potential of HAS3‐EVs (−40.8 mV) was also considerably more negative compared to MCF7‐EVs (−27.6 mV), indicating the presence of negatively charged HA (Figure 1I). Interestingly, the size of isolated MCF7‐EVs and HAS3‐EVs was similar when measured by nanoparticle tracking analysis, despite the HA‐decorated subpopulation of HAS3‐EVs (Figure 1A, B). Enzymatic digestion of HA with hyaluronidase also did not have effect on the size of the HAS3‐EVs (Figure S2). Characterisation of EVs was done according to the MISEV guidelines (Welsh et al. 2024). The expression of common EV markers ALIX, TSG101, CD63 and CD9, and morphology assessed by transmission electron microscopy were similar between MCF7‐EVs and HAS3‐EVs (Figure 1C–E).

### 3.2 CD44 and HA Are Required for Efficient HAS3‐EV Binding

**FIGURE 2:**
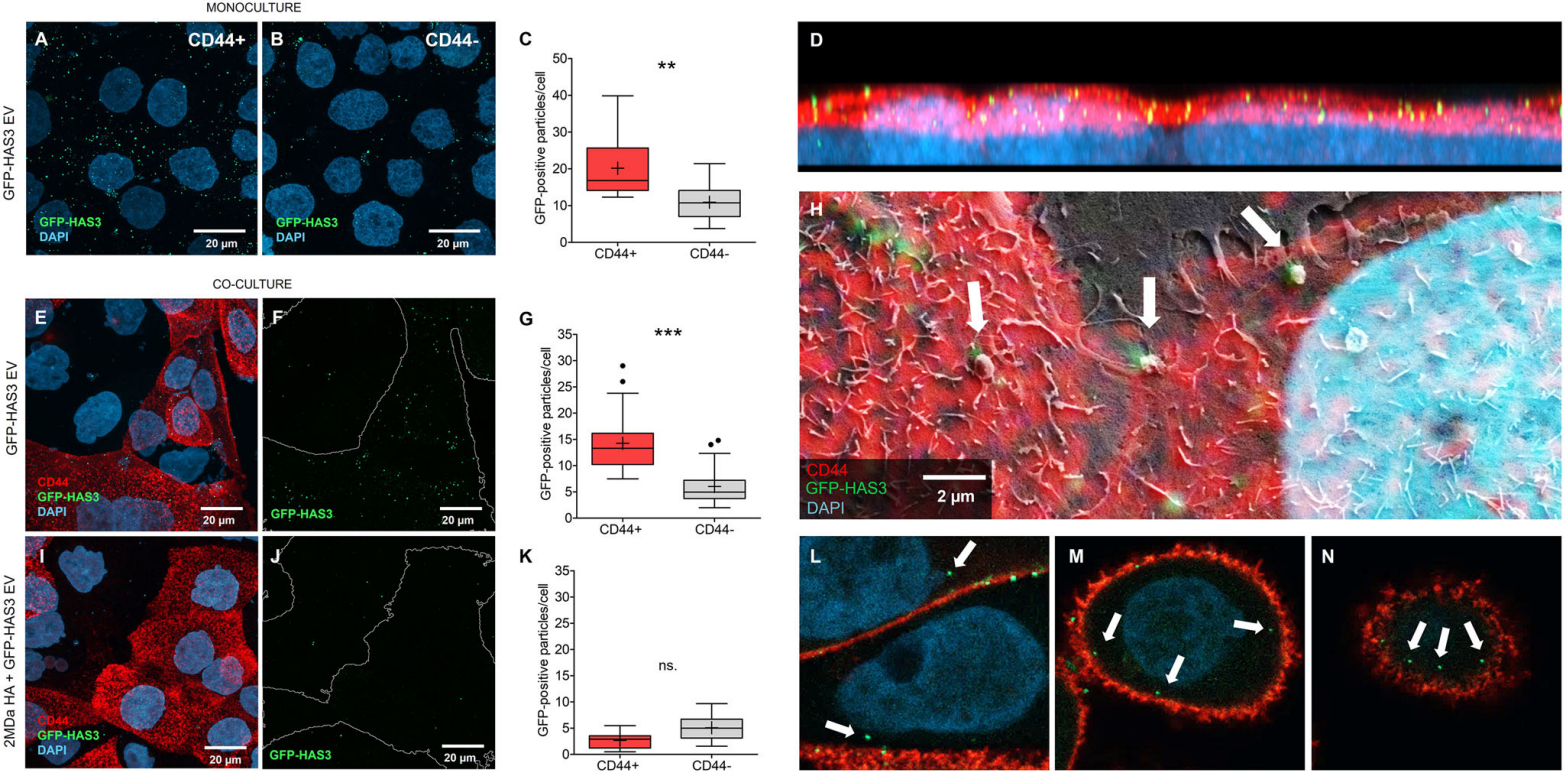
CD44‐expression enhances binding and uptake of HA‐decorated EVs. Confocal imaging of fixed GFP‐HAS3‐EV treated MKN74 CD44^+^ (A) and CD44^−^ (B) cells after 5 h incubation. (C) Quantification of GFP‐positive spots per cell in CD44^+^ and CD44^−^ cultures [*n* = 5 (a total of 334 CD44^+^ and 337 CD44^−^ cells), Mann–Whitney *U* test, ** *p* < 0.01]. (D) Side view of the 3D‐projection from CD44^+^ culture after 5 h treatment with GFP‐HAS3‐EVs. (E–G) MKN74 CD44^+^/CD44^−^ coculture after 5 h treatment with GFP‐HAS3‐EVs. Panel (F) outlines the area of CD44^+^ cells and the GFP‐HAS3‐EVs with a white line. (G) Quantification of GFP‐positive spots in CD44^+^ and CD44^−^ cells [*n* = 5 (a total of 263 CD44^+^ cells and 276 CD44^−^ cells), Mann–Whitney *U* test, *** *p* < 0.001]. (H) Correlative light and electron microscopy image shows vesicular structures (arrows) colocalising with GFP‐spots on the surface of CD44^+^ cells. (I–K) High molecular weight (2 MDa) HA pretreatment for CD44^+^/CD44^−^ coculture before addition of GFP‐HAS3‐EVs. A white line in panel (J) marks the area occupied by CD44^+^ cells. (K) Quantification of GFP‐positive spots per cell [*n* = 5 (a total of 224 CD44^+^ and 272 CD44^−^ cells), Mann–Whitney *U* test, ns. = not significant]. (L–N) Arrows point at internalised GFP‐HAS3‐EVs from single optical sections. EV, extracellular vesicle; HA, hyaluronic acid.

To assess whether HA receptor CD44 is required for HAS3‐EV interactions with cells, we utilised two MKN74 gastric cancer cell lines, one transfected with human CD44s (CD44^+^) and a mock‐transfected control cell line (CD44^−^). To compare HA‐decorated EV binding, both cell types were treated with HAS3‐EVs, and the number of GFP‐positive spots was quantified from 3D confocal microscopy image stacks (Figure 2). As anticipated, CD44^+^ cells bound significantly more (1.8‐fold increase) GFP‐HAS3‐EVs compared to CD44^−^ cells (Figure 2A–C).

To further investigate CD44‐dependent targeting of HAS3‐EVs, CD44^+^ and CD44^−^ cells were cocultured and treated with HAS3‐EVs. In this setting, an increased targeting (2.4‐fold increase compared to CD44−) of GFP‐HAS3‐EVs to CD44^+^ cells was observed (Figure 2E–G). To confirm that this interaction between HAS3‐EVs and cells was indeed CD44‐dependent, we attempted to oversaturate the CD44 receptors using either short HA‐oligomers (Figure S3) or high molecular weight HA (Figure 2I, K). Pretreatment with HA‐oligomers resulted in only a modest reduction in the number of GFP‐HAS3‐EVs bound to the CD44^+^ cells. However, pretreatment with high molecular weight HA nearly completely blocked the binding of GFP‐HAS3‐EVs to CD44^+^ cells, while the number of GFP‐HAS3‐EVs associated with CD44^−^ cells remained unchanged (Figure 2K).

Confocal microscopy and correlative light and electron microscopy analysis revealed that the majority of the GFP‐HAS3‐EVs appeared to remain bound to the plasma membrane rather than being internalised (Figure 2D, H). However, individual EVs were detected in the cytosol, mainly localised in the close proximity of the plasma membrane (Figure 2L–N).

To determine whether the HA already bound to CD44 on the cell surface limits the ability of HA‐decorated EVs to attach, we enzymatically removed surface HA by hyaluronidase (Figure S4). Under normal conditions, the CD44^+^ cells exhibited a thin HA coat, which was effectively removed following hyaluronidase treatment (Figure S4). Interestingly, removal of cell‐associated HA before incubation with EVs did not enhance HAS3‐EV binding, but instead it significantly reduced binding to levels comparable to those observed in CD44^−^ cells (Figure S5). Similarly, digestion of HA from HAS3‐EVs before incubation with cells did not increase their binding to CD44^−^ cells (Figure S5).

**FIGURE 3:**
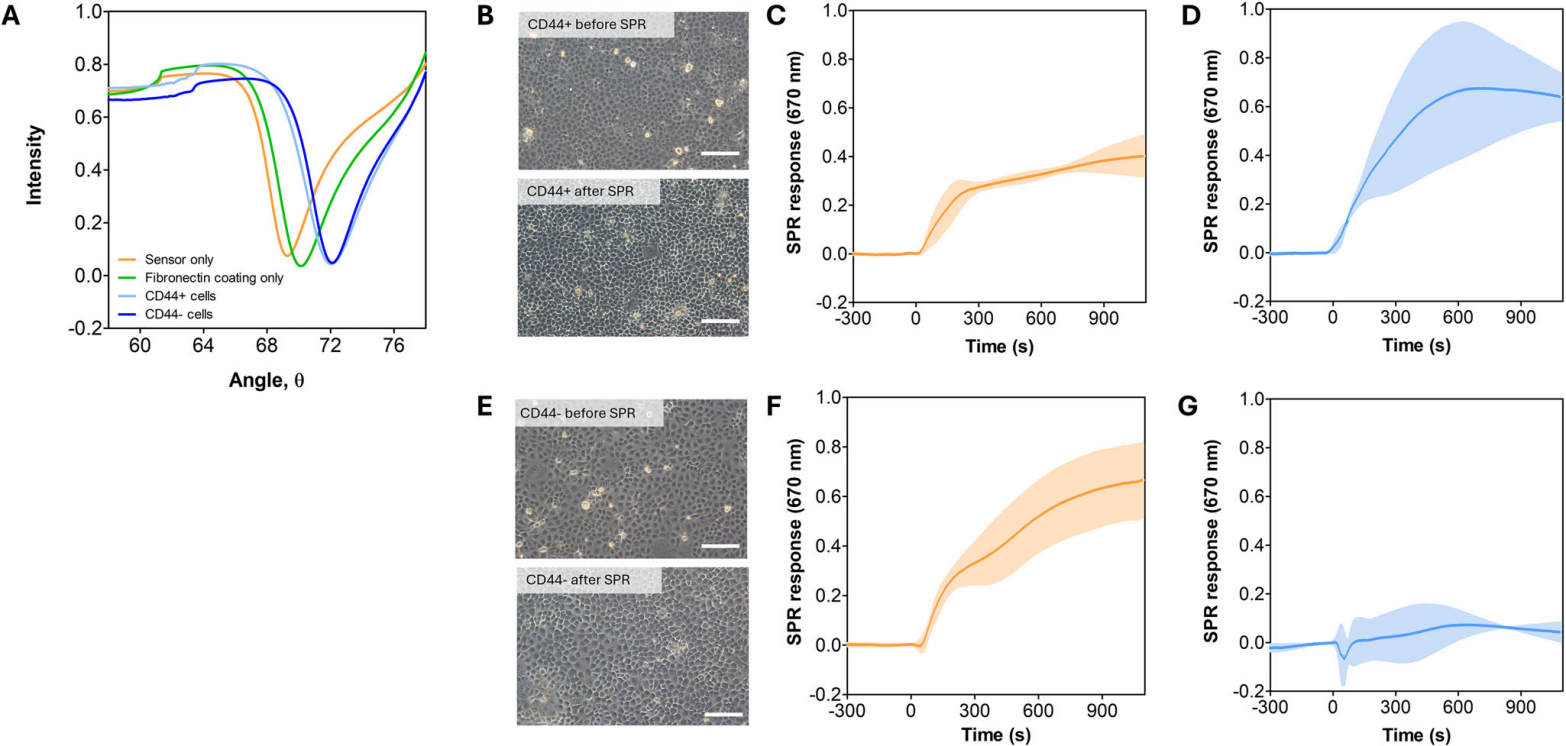
Label‐free MP‐SPR quantification of MCF7‐EV and HAS3‐EV interactions with CD44^+^ and CD44^−^ cells. (A) Representative SPR curve comparison between blank gold sensor, fibronectin coated sensor, CD44^+^ monolayer, and CD44^−^ monolayer. Phase contrast microscopy images of the (B) CD44^+^ and (E) CD44^−^ cell monolayers before and after the SPR measurement. Scale bars correspond to 200 µm. MP‐SPR signal of the interaction between MCF7‐EVs and (C) CD44^+^ cells or (F) CD44^−^ cells. Interaction between HAS3‐EVs with (D) CD44^+^ cells or (G) CD44^−^ cells. SPR‐curves with standard deviation from two (HAS3‐EVs with CD44^+^ cells) to three biological replicate measurements are shown. EV, extracellular vesicle; HA, hyaluronic acid.

Next, we investigated the interactions of MCF7‐EVs and HAS3‐EVs between CD44^+^ and CD44^−^ cells. A label‐free, multiparametric surface plasmon resonance method (Koponen et al. 2020) was adapted to quantitatively measure the interactions between the two types of EVs and MKN74‐cell monolayers (Figure 3). MCF7‐EVs showed a clear interaction with both CD44^+^ and CD44^−^ cells, with approximately 1.5‐fold higher signal observed with CD44^−^ cells (Figure 3C, F), suggesting that the interactions of these non‐HA‐coated MCF7‐EVs with target cells do not rely on CD44. As expected, HAS3‐EVs exhibited a high signal with CD44^+^ cells (Figure 3D), while a minimal signal was observed with CD44^−^ cells, indicating that HAS3‐EVs have a high affinity to CD44^+^ cells, but have little or no ability to interact with the CD44^−^ cells (Figure 3G).

### 3.3 Uptake Dynamics of MCF7‐EVs and HAS3‐EVs

**FIGURE 4:**
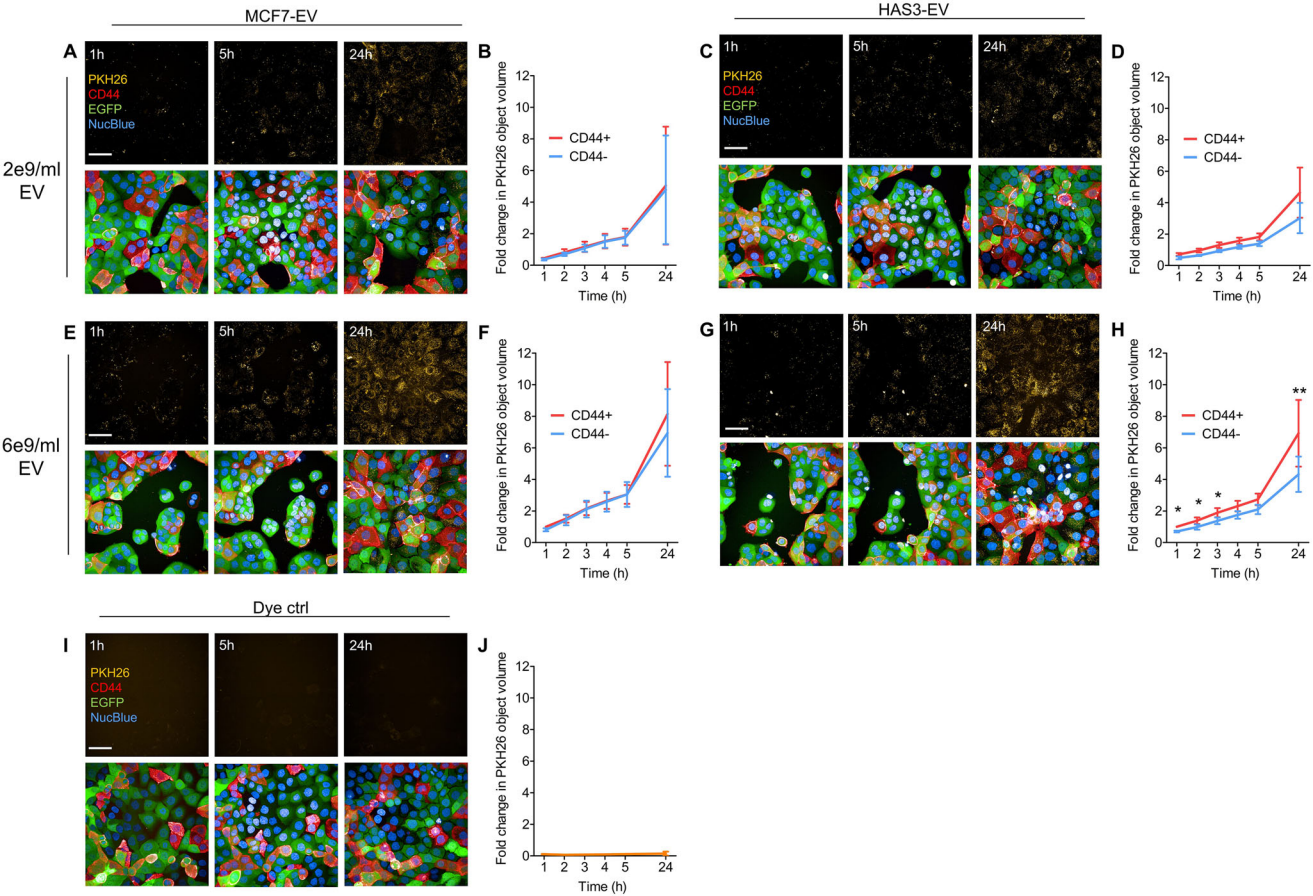
Time‐ and concentration‐dependent uptake of PKH26‐labelled MCF7‐EVs and HAS3‐EVs in CD44^+^/CD44^−^ cocultures. Maximum intensity projections and quantification of the uptake of 2e9/mL MCF7‐EVs (A, B) and HAS3‐EVs (C, D) in MKN74 cocultures at 1–5 h, and 24 h timepoints. Increased uptake was observed with higher 6e9/mL concentration of MCF7‐EVs (E, F) and HAS3‐EVs (G, H). Maximum intensity projections and quantification of the cultures treated with PKH26 dye control (I, J). Statistically significant differences in CD44^+^/CD44^−^ uptake per timepoint based on linear mixed model are marked with asterisks (*p < 0.05, **p < 0.01). For visualisation, mean ± SEM, normalised to CD44^+^ uptake at 1 h are presented, *n* = 3, 10 images from 2 wells/experiment. The scale bars represent a length of 50 µm. EV, extracellular vesicle; HA, hyaluronic acid.

Fluorescent labelling of EVs was utilised to compare the dynamics of uptake of MCF7‐EVs and HAS3‐EVs by the MKN74 cells. After labelling, both EV types retained their original size distribution and morphology, as confirmed by TEM (Figure S6). Using high‐content imaging, time‐dependent uptake of labelled EVs was monitored in live MKN74 CD44^+^/CD44^−^ cocultures over a 24‐h period. Two concentrations of labelled EVs were administered to the cells, and 3D confocal image stacks were captured at various time points. The amount of labelled EVs per cell type was then quantified from the images (Figure 4). The uptake of both MCF7‐EVs and HAS3‐EVs was concentration dependent in both CD44^+^ and CD44^−^ cells and increased over time. MCF7‐EV uptake was unaffected by CD44‐expression and was equal between CD44^+^ and CD44^−^ cells (Figure 4A, B, E, F). Expectedly, HAS3‐EVs showed a trend of increased targeting towards CD44^+^ cells at lower concentration (2e9/mL) (Figure 4C, D), and significantly increased targeting with higher concentration (6e9/mL) across 1, 2, 3 and 24 h timepoints (Figure 4G, H). Increased targeting was particularly evident after 24‐h incubation with the labelled EVs, indicating CD44‐mediated binding of the HA‐decorated subpopulation. The overall CD44^+^/CD44^−^ ratio (1.39, *p* < 0.0001) at 6e9/mL concentration remained similar across the different timepoints. The contrast with the GFP‐HAS3‐EVs likely reflects the presence of a labelled subpopulation of EVs lacking the HA‐decoration, possibly originating from the exosomal pathways (Mathieu et al. 2021), while the HA‐decorated GFP‐HAS3‐EVs originate primarily from the plasma membrane.

### 3.4 Endocytosis Mechanisms of MCF7‐EVs and HAS3‐EVs

Next, the potential endocytosis pathways involved in the uptake of MCF7‐EVs and HAS3‐EVs were investigated. Free HA has been suggested to be endocytosed via CD44 (Knudson et al. 2002) or HARE (Pandey and Weigel 2014) receptors, utilising various endocytic pathways, including lipid raft, caveolae and clathrin‐mediated pathways, as well as macropinocytosis (Racine et al. 2012). Same endocytosis pathways have also been suggested for EV internalisation (Mulcahy et al. 2014). Therefore, commonly used inhibitors for the major endocytosis pathways were selected to inhibit lipid rafts (MβC), clathrin‐mediated endocytosis (chlorpromazine) and macropinocytosis (EIPA). Additionally, free high molecular weight HA, which was previously shown to decrease the binding of GFP‐HAS3‐EVs, was used to block HA‐CD44 interactions.

**FIGURE 5:**
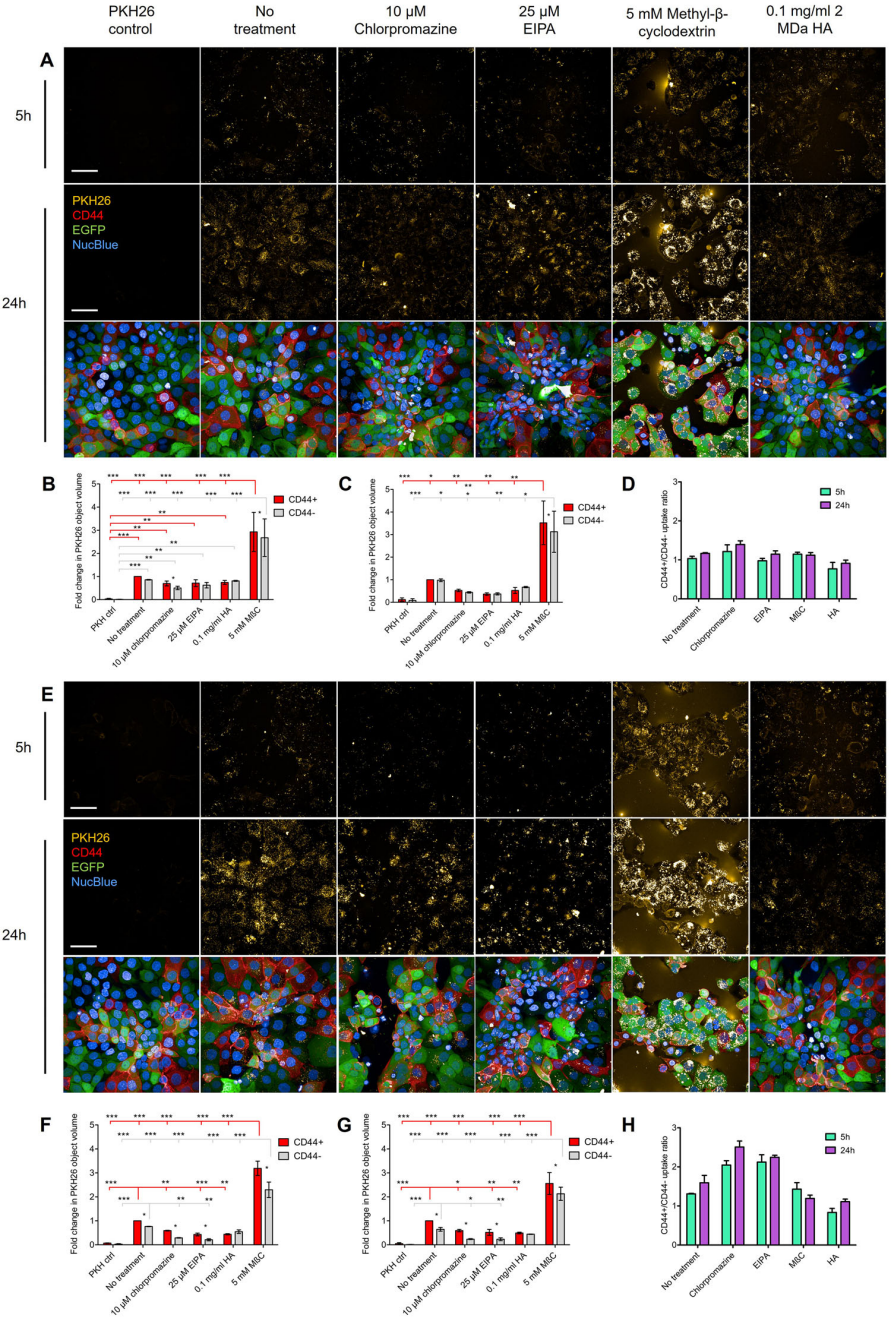
MCF7‐EV and HAS3‐EV uptake in the presence of endocytosis inhibitors. MKN74 cocultures were pretreated with MβC, Chlorpromazine, EIPA or high molecular weight HA before the addition of PKH26‐labelled EVs. The EV uptake was imaged in live cells for 5 h at 1‐h intervals, and a final timepoint was imaged 24 h after the addition of EVs. Maximum intensity projections of MKN74 cocultures treated with the endocytosis inhibitors and PKH26‐labelled (A) MCF7‐EVs, (E) HAS3‐EVs. PKH26 signal is shown from 5 and 24 h timepoints and a merged image from MKN74 coculture showing the morphology of the cells at the 24 h timepoint. Quantification of PKH26‐labelled (B, C) MCF7‐EV, (F, G) HAS3‐EV uptake into CD44^+^ and CD44^−^ cells at 5 and 24 h timepoints [*n* = 3 (10 images from 2 wells/experiment), mean normalised to CD44^+^ ctrl, SEM]. Statistically significant differences from linear mixed model within conditions, and between treatments (red line = CD44+, grey line = CD44^−^) are marked with asterisks (*p<0.05, **p<0.01, ***p<0.001). The fold‐change of EV uptake into CD44^+^ cells compared to CD44^−^ cells for (D) MCF7‐EVs and (H) HAS3‐EVs based on the quantified uptake. The scale bars represent a length of 50 µm. EV, extracellular vesicle; HA, hyaluronic acid.

MKN74 cocultures were pre‐treated with the endocytosis pathway inhibitors, before addition of PKH26‐labelled MCF7‐EVs or HAS3‐EVs. The cell cultures were then imaged live for 5 h at 1 h intervals and a final timepoint was imaged at 24 h after addition of the EVs (Figure 5, Figures S7, S8). Uptake of MCF7‐EVs was decreased by chlorpromazine and EIPA during the first 5‐h period after EV addition in both CD44^+^ and CD44^−^ cells, with a similar, but not statistically significant level of inhibition in both cell types (Figure 5A, B). After 24 h, the overall decrease in uptake was less pronounced compared to the inhibitor‐free control, but consistent between the two cell types, apart from chlorpromazine which significantly decreased uptake into CD44^−^ cells compared to CD44^+^ cells (Figure 5C). In contrast, treatment with MβC led to a significant increase in MCF7‐EV uptake at all timepoints in both CD44^+^ and CD44^−^ cells. However, the cultures were also characterised by accumulation of the PKH26 label and long‐term treatment with MβC also had a drastic effect on the morphology of the cells (Figure 5A). Similar increase of PKH26‐labelled EV uptake was not seen with breast epithelial and breast cancer cells (Figure S9). Both chlorpromazine and EIPA significantly reduced the uptake of HAS3‐EVs at 24 h (Figure 5E–G), but a significantly increased uptake of HAS3‐EVs by CD44^+^ cells was still observed in the presence of the inhibitors. Treatment with MβC majorly increased the HAS3‐EV uptake into both cell types. Interestingly, a trend of increased uptake ratio (CD44^+^ to CD44^−^cells) in the presence of chlorpromazine and EIPA was observed with HAS3‐EVs (Figure 5H), but not with MCF7‐EVs (Figure 5D).

To assess the role of CD44‐mediated binding, high molecular weight hyaluronan (HA) was added to saturate CD44 receptors (Figure 5). Surprisingly, HA treatment seemingly decreased the uptake of both MCF7‐EVs and HAS3‐EVs by 40%–70%, independent of the CD44 expression of the cells. However, the effect was significant only in CD44^+^ cells at both 5 and 24 h timepoints.

### 3.5 Multiparametric Surface Plasmon Resonance‐Based CD9‐Antibody Capture and Plasma Protein Corona Formation of EVs

Plasma‐derived biomolecular corona formation on the surface of EVs has been recently implicated in affecting their uptake and biodistribution (Dietz et al. 2023; Liam‐Or et al. 2024; Musicò et al. 2023). However, no studies currently exist on how specific EV surface components influence corona formation. Studies on HA‐decorated synthetic nanoparticles have shown differences in corona protein adsorption (Almalik et al. 2017; Kari et al. 2020). Therefore, we investigated how HA‐decoration affects plasma‐derived corona formation in EVs.

MP‐SPR‐based method, previously used to study protein corona formation on liposomes (Kari et al. 2017), was adapted to investigate corona formation on EVs. First, EVs were captured and immobilised onto avidin‐coated biosensors with biotinylated CD9‐antibody. CD9 was selected as the capture antibody based on its abundant expression in both MCF7‐EVs and HAS3‐EVs as demonstrated by western blots (Figure 1C), and its predominant localisation on the plasma membrane of MCF7‐cells (Figure S10). CD9 also colocalised with GFP‐HAS3 in the filopodia‐like protrusions (Figure S10). The localisation of CD9 in MCF7‐cells was also in line with previously reported literature (Fan et al. 2023).

**FIGURE 6:**
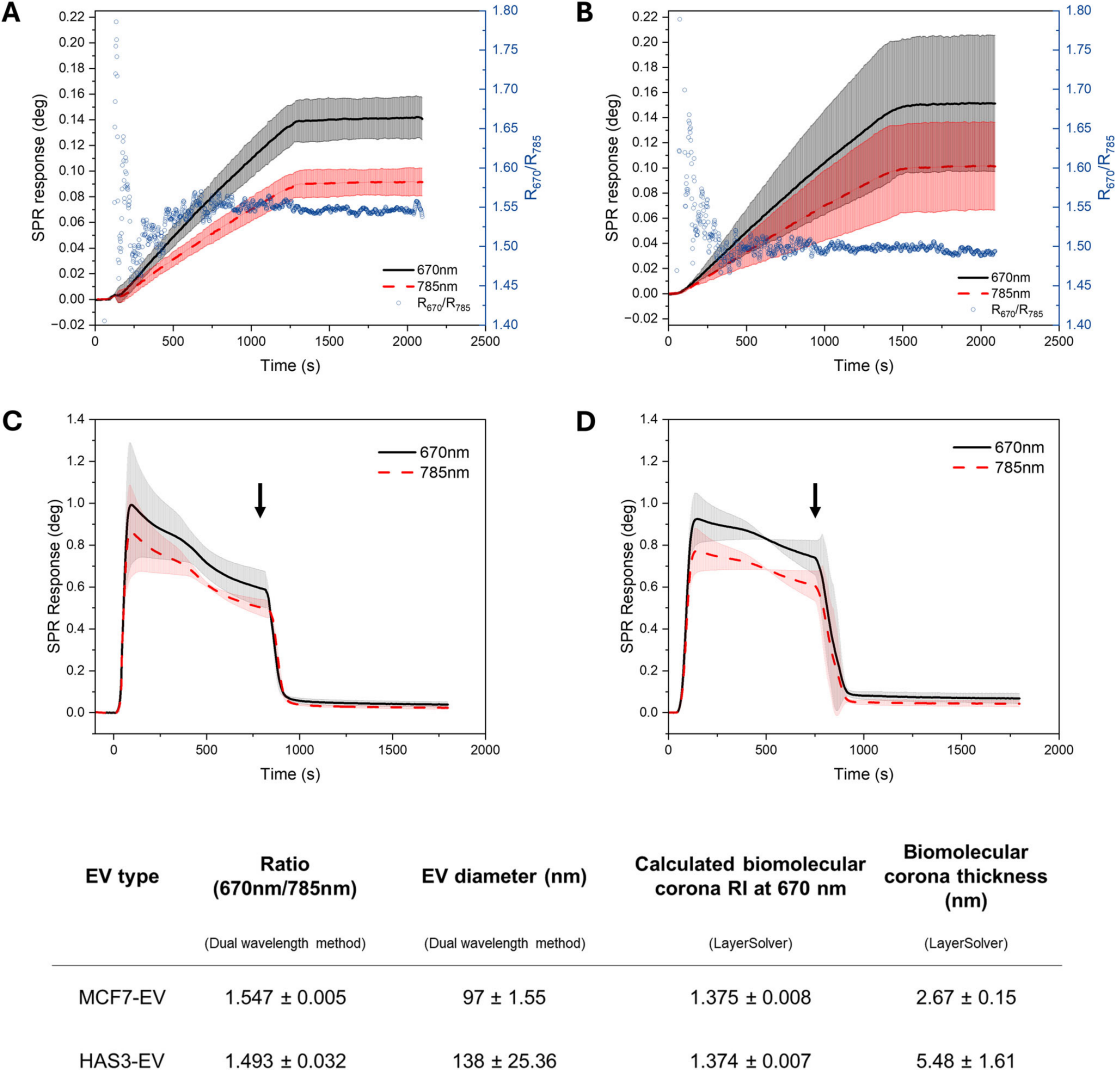
MP‐SPR based CD9‐antibody capture of EVs and plasma‐derived corona formation. SPR responses with 670 nm (line) and 785 nm lasers (dashed line) and the ratio of the two wavelengths (dots) from CD9‐antibody captured MCF7‐EVs (A) and HAS3‐EVs (B) over 30‐min injections of EVs (*n* = 3, STDEV). After EV capture, EV‐depleted plasma was injected to the system, causing a sharp increase in the SPR‐signal, followed by sharp decrease after washing with PBS (indicated with arrows). The corona refractive index and thickness were calculated from the remaining tail signal. SPR responses with 670 nm (line) and 785 nm lasers (dashed line) for plasma injection and protein corona formation on MCF7‐EVs (C) and HAS3‐EVs (D) (*n* = 3, STDEV). The average ratio of the signals measured at 670 and 785 nm for the dual‐wavelength method to calculate the EV diameters, and refractive index (RI) and thickness of the protein corona determined with LayerSolver are shown in the table. EV, extracellular vesicle; HA, hyaluronic acid.

We first confirmed that the EVs are unable to bind to the avidin‐only coated biosensors. Injection of MCF7‐EVs or HAS3‐EVs did not result in any notable signal compared to the baseline (Figure S11A, B). Following EV injection to the CD9 antibody coated sensors, an increasing signal that reached an equilibrium was observed, implying successful EV capture and immobilisation to the biosensors (Figure 6A, B). The formed EV layer thickness, corresponding to the average diameter of the immobilised EVs, was calculated using the ratio of the signal from the 670 and 785 nm wavelengths. The average diameter of the CD9 antibody‐captured HAS3‐EVs (138 ± 25.36 nm) was notably larger than the diameter of MCF7‐EVs (97 ± 1.55 nm) (Figure 6).

After successful capture of the EVs, EV‐depleted human plasma was injected into the system to result in protein corona formation. Plasma exposure caused a substantial increase in the SPR signal due to change in the refractive index compared to the PBS buffer of EVs (Figure 6C, D). After the plasma injection, PBS was reintroduced for washing, which caused a sharp decline in the signal. The remaining signal implied that a thin coronal layer formed on the surface of the CD9‐captured EVs. The refractive index and thickness of the corona layer were determined using LayerSolver software. Interestingly, the thickness of the corona formed around HAS3‐EVs (5.48 ± 1.61 nm) was over twofold greater compared to the corona of MCF7‐EVs (2.67 ± 0.15 nm) (Figure 6). Because the EV capture signal was used as the baseline for calculating corona formation, the calculated corona thickness was not influenced by the presence of HA on the EV surface. Low SPR‐signal was observed after plasma injection and washing with avidin‐coated sensors (Figure S11C), indicating low affinity of plasma proteins to the sensor without the immobilised EVs.

### 3.6 Mass Spectrometry Analysis of EVs and the Formed Protein Corona

To analyse the protein content of both the nascent EVs and EVs with the plasma‐derived corona, the MP‐SPR captured EVs were detached from the sensor and collected for mass spectrometry analysis. Successful capture of the CD9‐positive subpopulation of EVs was confirmed with a comparative analysis of HAS3‐EVs that had not undergone the MP‐SPR capture. We found several proteins that were significantly increased or decreased in abundance, indicating that MP‐SPR‐captured HAS3‐EVs represented a distinct subpopulation (Figure S12). The proteome of nascent EVs was first analysed to distinguish the intrinsic EV‐proteins from the plasma‐acquired corona proteins. Mass spectrometry analysis of nascent MP‐SPR‐captured EVs identified 84 proteins in MCF7‐EVs and 168 proteins in HAS3‐EVs (Figure S13, Table S1). The reason for low number of identified proteins was most likely due to technical limitation related to the small capture area of the MP‐SPR system, which yielded a low amount of protein after release of the captured EVs. Regardless, considerable proportion of the identified proteins (57) were shared between the two EV types.

**FIGURE 7:**
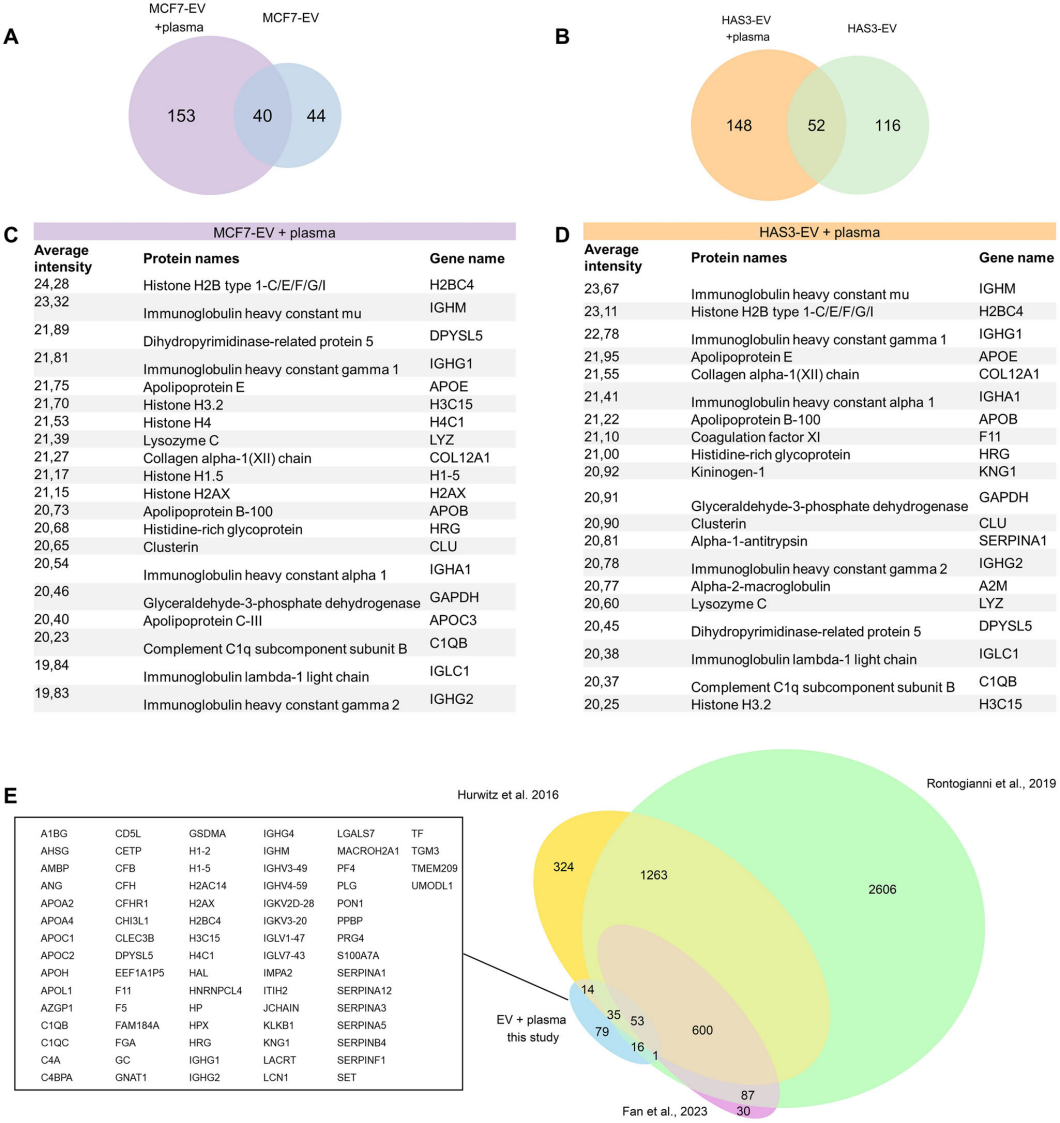
Protein composition and possible corona proteins of plasma‐exposed MCF7‐EVs and HAS3‐EVs. Venn diagrams of proteins detected in plasma‐exposed and nascent (A) MCF7‐EVs and (B) HAS3‐EVs. The Top 20 most enriched proteins are shown for plasma‐exposed MCF7‐EVs (C) and plasma‐exposed HAS3‐EVs (D) based on average LC‐MS/MS intensity from three biological replicate samples. (E) Comparison of proteins detected from plasma‐exposed EVs in this study with protein composition of nascent MCF7‐EVs from two previous studies (Fan et al. 2023; Hurwitz et al. 2016), and multiple nascent breast cancer cell line EVs (Rontogianni et al. 2019). Text box shows the 79 proteins detected only in the plasma‐exposed EVs in this study. EV, extracellular vesicle; HA, hyaluronic acid.

After plasma exposure, an increase in total protein content (Table S2) was detected both in MCF7‐EVs and HAS3‐EVs, indicating association of plasma‐derived proteins with the EVs. Mass spectrometry analysis revealed an increased number of proteins compared to nascent EVs, with a total of 193 detected proteins in plasma‐exposed MCF7‐EVs and 200 proteins detected in plasma‐exposed HAS3‐EVs (Figure 7A, B, Table S2). In MCF7‐EVs, 40 proteins were shared between the nascent and the plasma‐exposed EVs (Figure 7A) and in HAS3‐EVs, 52 shared proteins were identified between the nascent and the plasma‐exposed EVs (Figure 7B).

The most enriched proteins common for both plasma‐exposed MCF7‐EVs and HAS3‐EVs included immunoglobulin chains IGHM, IGHG1, IGHA1, IGLC1 and IGHG2, and apolipoproteins APOE and APOB, as well as complement factor C1QB (Figure 7C, D). The Top 20 most enriched proteins in plasma exposed MCF7‐EVs also included multiple histone proteins, while HAS3‐EVs were enriched with protease inhibitors (SERPINA1 and A2M), and proteins related to blood coagulation (F11 and KNG1).

Due to the low number of proteins detected in the nascent EV samples, the plasma‐exposed EV proteome was compared to three large proteome datasets from MCF7‐EVs (Fan et al. 2023; Hurwitz et al. 2016) or multiple breast cancer cell line EVs including MCF7‐EVs (Rontogianni et al. 2019) (Figure 7E, Table S4). Out of the 203 unique proteins detected from either plasma‐exposed MCF7‐EVs or plasma‐exposed HAS3‐EVs, 79 proteins were not found in any of the three large MCF7‐EV proteome sets, which implied plasma‐derived origin of those proteins and their possible association with the EV corona. Several proteins that are part of commonly reported protein families associated with plasma‐derived protein corona of EVs (Tóth et al. 2021) were among the 79 unique proteins. These included apolipoproteins (APOA2, APOA4, APOC1, APOC2, APOH and APOL1), complement system proteins (C1QB, C1QC, C4A and C4BPA), coagulation factors (F11 and F5), immunoglobulin chains (IGHG1, IGHG2, IGHG4, IGHM, IGHV3‐49, IGHV4‐59, IGKV2D‐28, 1GKV3‐20, IGLV1‐47 and IGLV7‐43) and serine protease inhibitors (SERPINA1, SERPINA12, SERPINA3, SERPINE5, SERPINB4 and SERPINF1).

**FIGURE 8:**
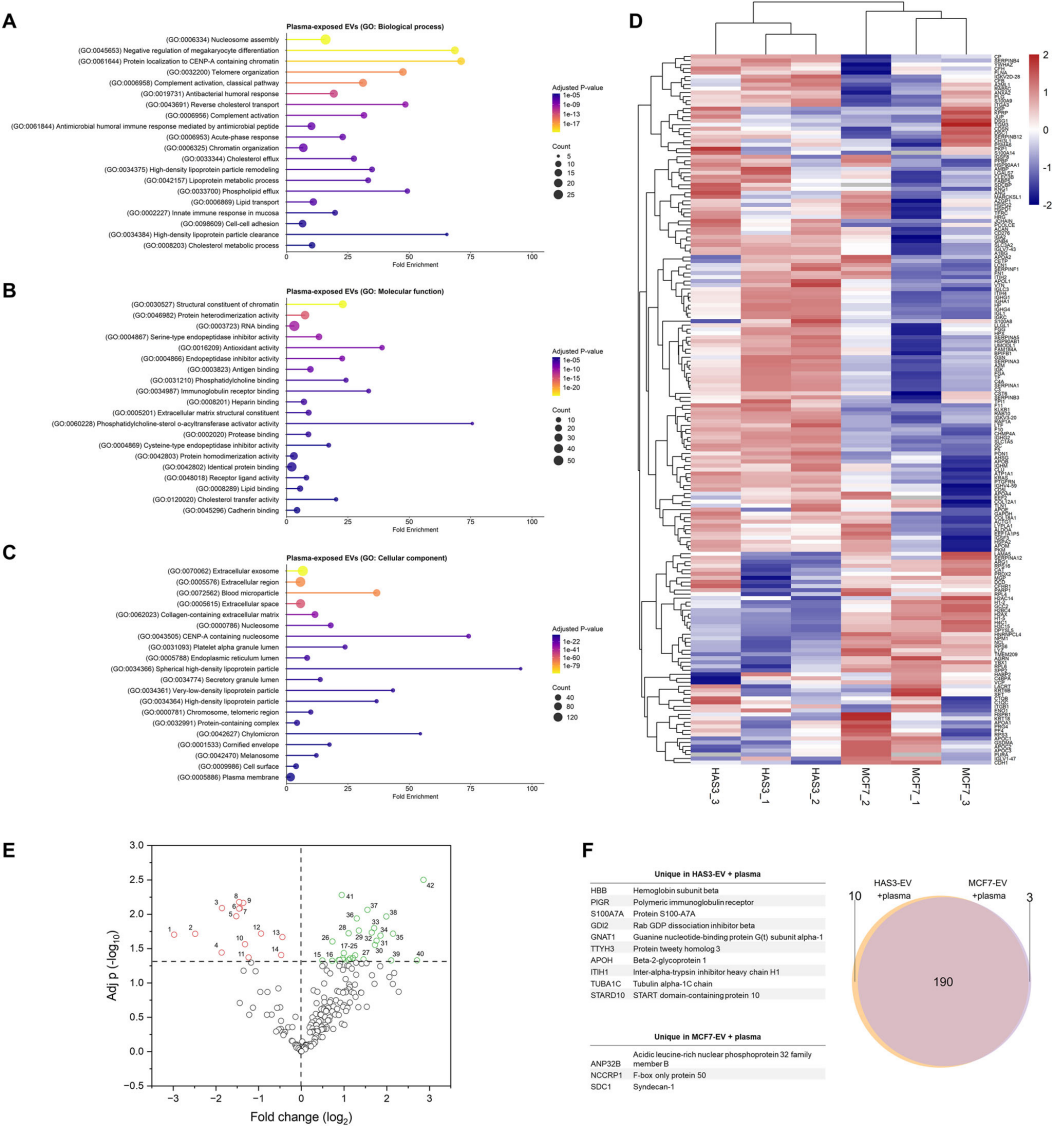
Altered protein corona composition in HA‐decorated EVs. Functional enrichment analysis was performed on the shared proteome of the plasma‐exposed EVs. The 20 most enriched Gene Ontology (GO) terms based on Benjamini adjusted *p* value are shown for (A) GO: Biological process, (B) GO: Molecular function and (C) GO: Cellular component. (D) Heatmap of the 190 proteins shared by plasma‐exposed MCF7‐EVs and HAS3‐EVs. Log_2_‐transformed intensity values from three biological replicates per EV type were used for heatmap generation. The data were *z* score normalised and centred across rows to highlight relative abundance patterns between samples and hierarchical clustering for rows and columns was performed. (E) Volcano plot showing statistically significant changes in protein expression in plasma‐exposed HAS3‐EVs compared to plasma‐exposed MCF7‐EVs. Proteins with an adjusted (−log10) *p* value of 1.30 (marked with a dashed line) were considered significantly differently enriched. Numerically labelled significant proteins are listed in Table 2. (F) Venn diagram of identified common and unique proteins between the plasma‐exposed HAS3‐EVs and plasma‐exposed MCF7‐EVs with the unique proteins listed on the side. EV, extracellular vesicle; HA, hyaluronic acid.

Gene Ontology functional enrichment analysis for the shared plasma‐exposed EV proteome indicated significant enrichment in plasma‐derived proteins. Most significantly enriched biological processes were related to cholesterol and lipid transport, complement activation and lipoprotein metabolism (Figure 8A), as well as chromatin binding, explained by enrichment of histone proteins in the samples. Significant enrichment of endopeptidase inhibitor activity, antioxidant activity and immunoglobulin receptor binding were among the most enriched molecular functions, with phosphatidylcholine‐sterol‐*o*‐acyltransferase activator activity having the highest fold enrichment (Figure 8B). For cellular component, extracellular exosome was the most significant term, with highest fold enrichment being related to lipoprotein particles, indicating EV interaction with lipoproteins (Figure 8C). All statistically significant GO terms are listed in Table S3. Out of the whole proteome of plasma‐exposed MCF7‐EVs and HAS3‐EVs, 190 of the detected proteins were shared between the two EV‐types (Figure 8F). However, hierarchical clustering of proteomic profiles showed a distinct clustering patterns between the two sample types (Figure 8D).

Differential expression analysis was performed for the plasma‐exposed EVs to compare the enrichment of proteins between HAS3‐EVs and MCF7 EVs. A total of 42 proteins (Figure 8B, listed in Table 2) were significantly differentially enriched in HAS3‐EVs compared to MCF7‐EVs. Among those, 14 proteins had a reduced abundance (negative log_2_ fold change) while the remaining 28 proteins had an increased abundance (positive log_2_ fold change) in HAS3‐EVs. The proteins with reduced abundance were mainly histones or other proteins related to the nucleus, and were mainly found also in the nascent EVs either in this or in previous studies, with the exception of two proteins (TMEM209 and HNRNPCL4). The 28 significantly enriched proteins in contrast were majorly plasma‐derived and protein corona associated, with enrichment in immunoglobulin heavy constants, complement factors C3 and C4A, fibrinogen alpha chain, coagulation factor V and serine protease inhibitors SERPINA1 and SERPINA3. Other notable significantly enriched proteins included HA‐binding protein Inter‐alpha‐trypsin inhibitor heavy chain H4 (ITIH4), serotransferrin (TF) and Vitamin D‐binding protein (GC). Twelve of the 28 proteins were not identified in nascent EVs in either this or previous studies, as detailed in Table 2. Those included several proteins associated with a protein corona.

**TABLE 2:** Statistically significant differentially enriched proteins in plasma‐exposed HAS3‐EVs compared to plasma‐exposed MCF7‐EVs.

Numeric label from Figure 8E	Gene name	Protein name	Uniprot accession	Fold change (log2)	Adj. *p* value	EV protein (previous studies)	EV protein (this study)
**1**	**TMEM209**	**Transmembrane protein 209**	**Q96SK2**	**−2**.**977**	**1.704**		
2	YBX1	Y‐box‐binding protein 1	P67809	−2.484	1.715	X	
**3**	**HNRNPCL4**	**Heterogeneous nuclear ribonucleoprotein C‐like 4**	**P0DMR1**	**−1.858**	**2.09**		
4	NPM1	Nucleophosmin	P06748	−1.866	1.443	X	X
5	H4C1	Histone H4	P62805	−1.523	1.971	X	X
6	DPYSL5	Dihydropyrimidinase‐related protein 5	Q9BPU6	−1.438	2.088		X
7	H1‐5	Histone H1.5	P16401	−1.456	2.177		X
8	H3C15	Histone H3.2	Q71DI3	−1.455	2.083	X	X
9	H2AX	Histone H2AX	P16104	−1.357	2.166		X
10	GCC2	GRIP and coiled‐coil domain‐containing protein 2	Q8IWJ2	−1.319	1.564	X	
11	RPS6	Small ribosomal subunit protein eS6	P62753	−1.233	1.371	X	
12	NCL	Nucleolin	P19338	−0.942	1.719	X	X
13	H1‐2	Histone H1.2	P16403	−0.443	1.668		X
14	AGRN	Agrin	O00468	−0.471	1.407	X	X
15	FABP5	Fatty acid‐binding protein 5	Q01469	0.494	1.327	X	X
16	IGKC	Immunoglobulin kappa constant	P01834	0.723	1.323	X	
17	PTGFRN	Prostaglandin F2 receptor negative regulator	Q9P2B2	0.995	1.435	X	
**18**	**FGA**	**Fibrinogen alpha chain**	**P02671**	**1.261**	**1.402**		
19	IGHG1	Immunoglobulin heavy constant gamma 1	P01857	0.972	1.359		X
**20**	**A1BG**	**Alpha‐1B‐glycoprotein**	**P04217**	**1.14**	**1.356**		
21	ITGA2	Integrin alpha‐2	P17301	1.205	1.363	X	
22	CHMP4A	Charged multivesicular body protein 4a	Q9BY43	0.977	1.338	X	
23	IGHA1	Immunoglobulin heavy constant alpha 1	P01876	0.87	1.33	X	
24	IGHG4	Immunoglobulin heavy constant gamma 4	P01861	1.113	1.336		X
25	ITIH4	Inter‐alpha‐trypsin inhibitor heavy chain H4	Q14624	0.894	1.332	X	
**26**	**AHSG**	**Alpha‐2‐HS‐glycoprotein**	**P02765**	**0.729**	**1.604**		
**27**	**SERPINA3**	**Alpha‐1‐antichymotrypsin**	**P01011**	**1.457**	**1.342**		
**28**	**C4A**	**Complement C4‐A**	**P0C0L4**	**1.107**	**1.723**		
29	ATP1A1	Sodium/potassium‐transporting ATPase subunit alpha‐1	P05023	1.346	1.762	X	X
30	SLC3A2	Amino acid transporter heavy chain SLC3A2	P08195	1.725	1.554	X	X
31	A2M	Alpha‐2‐macroglobulin	P01023	1.761	1.615	X	
32	C3	Complement C3	P01024	1.648	1.734	X	
33	SLC1A5	Neutral amino acid transporter B(0)	Q15758	1.705	1.799	X	
**34**	**SERPINA1**	**Alpha‐1‐antitrypsin**	**P01009**	**1.854**	**1.689**		
**35**	**TF**	**Serotransferrin**	**P02787**	**2.147**	**1.718**		
**36**	**F5**	**Coagulation factor V**	**P12259**	**1.298**	**1.939**		
37	RAB10	Ras‐related protein Rab‐10	P61026	1.548	2.064	X	
38	RAP1A	Ras‐related protein Rap‐1A	P62834	1.99	1.967	X	
**39**	**GC**	**Vitamin D‐binding protein**	**P02774**	**2.102**	**1.328**		
40	S100A9	Protein S100‐A9	P06702	2.709	1.329	X	X
**41**	**IGHG2**	**Immunoglobulin heavy constant gamma 2**	**P01859**	**0.946**	**2.28**		
42	S100A16	Protein S100‐A16	Q96FQ6	2.861	2.5	X	X

*Note*: Proteins not identified in nascent EVs in this study, Fan et al. 2023, Rontogianni et al. 2019 or Hurwitz et al. 2016 are bolded.

Between plasma‐exposed MCF7‐EVs and HAS3‐EVs, three unique proteins were identified from MCF7‐EVs and 10 unique proteins from HAS3‐EVs (Figure 8F). Several of the unique proteins in HAS3‐EVs appeared to originate from plasma, as protein S100‐A7A (S100A7A) and beta‐2‐glycoprotein (APOH) are not detected in the nascent MCF7‐EVs (Figure 7E). Along with the enrichment of HA‐binding protein ITIH4, ITIH1 was also identified as a unique protein for plasma‐exposed HAS3‐EVs.

### 3.7 Bovine Serum and Human Plasma Derived Corona in HAS3‐EV Uptake

**FIGURE 9:**
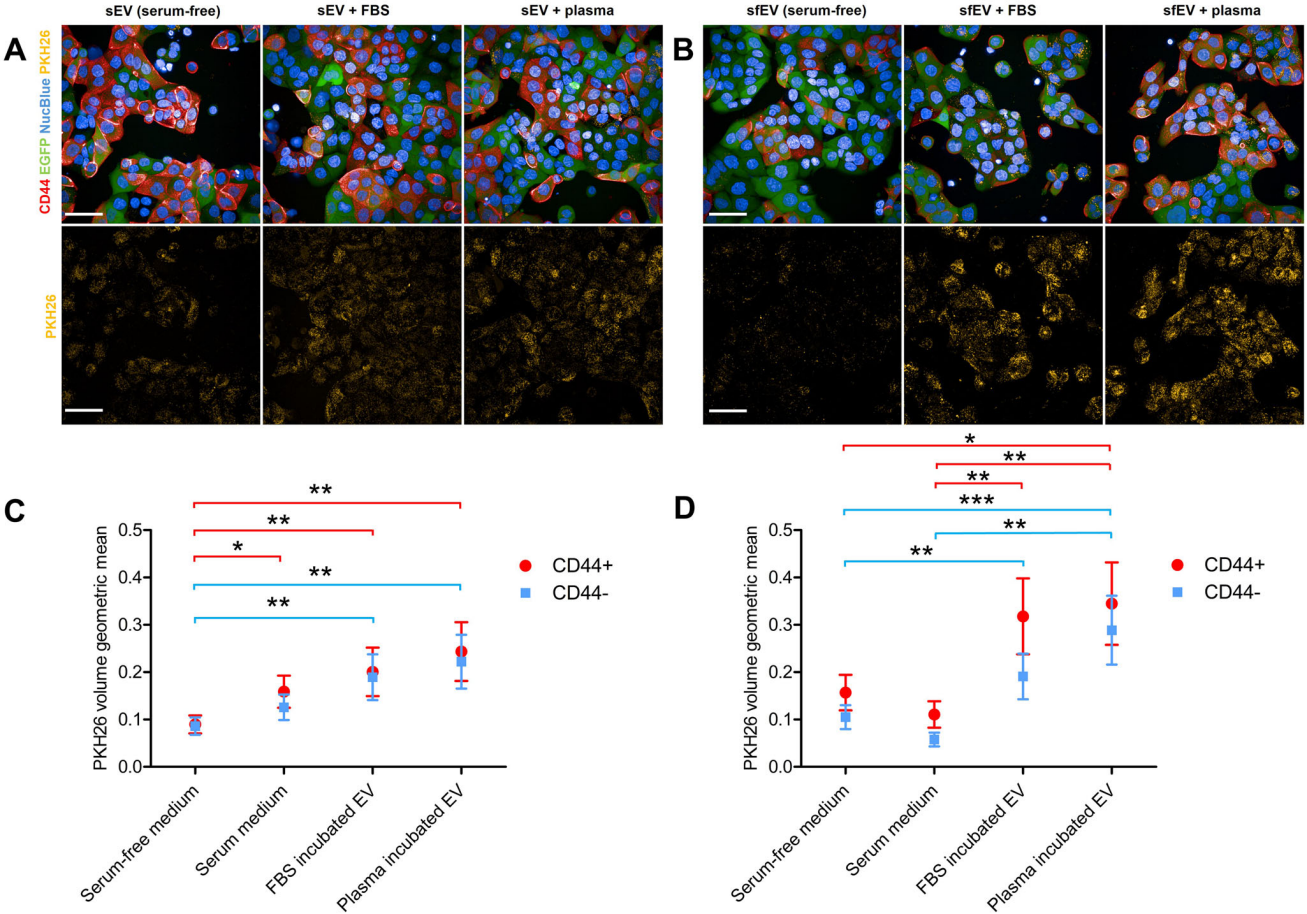
Uptake of HAS3‐EV with and without protein corona. HAS3‐EVs isolated from serum‐containing cultures (sEV) and serum‐free cultures (sfEV) were administered to MKN74 cocultures in serum‐containing or serum‐free medium or incubated with EV‐depleted FBS or plasma before administration in serum‐free medium. Maximum intensity projections of MKN74 cocultures with labelled sEVs (A) or sfEVs (B) under the different conditions. Geometric means and SEM of quantified PKH26 volume from sEVs (C) and sfEVs (D), *n* = 4 (except sEV + FBS, sEV + plasma *n* = 2), 5–10 images from 2 wells/experiment. Statistically significant differences from the linear mixed model are marked with asterisks (*p < 0.05, **p < 0.01, ***p < 0.001, red line = between CD44^+^, blue line = between CD44^−^). Scale bars: 50 µm. EV, extracellular vesicle; HA, hyaluronic acid.

To examine whether the protein corona of HAS3‑EVs induced by serum affects their interactions with target cells, we performed the high content screening uptake assays both in the presence and absence of serum in the cell culture medium, with EVs produced and isolated either in the presence of serum, or in serum‐free conditions. HAS3‐EVs were isolated from serum‐containing (sEVs) and serum‐free cultures (sfEVs), labelled with PKH26 and incubated with MKN74 cocultures with or without serum. sEV uptake was slightly increased in serum‐containing cultures, compared to serum‐free cultures (Figure 9C), while sfEVs showed a trend of decreased uptake in serum‐containing cultures (Figure 9D).

As we reasoned that EV isolation by ultracentrifugation might reduce the protein corona formed in cell culture, we attempted to establish a corona for sEVs and sfEVs by incubating pre‐labelled EVs either with EV‐depleted FBS or EV‐depleted human plasma prior to the uptake experiments. After the corona establishment, a significantly increased PKH26 signal was observed with both EV types (Figure 9A–D), although in sfEVs it was more prominent (Figure 9B,D). The increase was significant in both CD44^+^ and CD44^−^ cells, with slightly increased CD44^+^/CD44^−^ uptake ratio between the cell types with sfEVs.

## 4 Discussion

Understanding how surface molecules facilitate EV targeting and internalisation is fundamental in EV biology. The EV surface is molecularly complex and heterogenous with numerous factors affecting the specificity of EV targeting and uptake (Hallal et al. 2022). Among surface molecules, adhesion receptors such as integrins are commonly reported to facilitate EV binding to target cells through interactions with ECM components (Elsharkasy et al. 2025; Isogai et al. 2025). Recently, increasing attention has been directed towards the EV surface glycans—complex carbohydrates attached to membrane lipids, or proteins through post‐translational modification (Reily et al. 2019). Glycosaminoglycans like HA or heparan sulphate are a specific class of glycans with long, linear polysaccharide chains. Heparan sulphate proteoglycans on the surface of target cells act as receptors and bind multiple ligands, facilitating uptake of EVs (Christianson et al. 2013; Song et al. 2023). Although HA on the plasma membrane has not been directly linked to EV uptake, HA on the surface of EVs can enhance targeting to CD44‐overexpressing cells. The present study demonstrates that CD44 is necessary for efficient binding of HA‐decorated EVs, as CD44‐deficient cells exhibited minimal binding capability.

CD44 is a widely used target for HA‐decorated nanocarriers and shows promise for EV targeting. Although broadly expressed in normal tissues, CD44 levels are notably increased in many tumours, including lung (Hu et al. 2018), pancreatic (Zhao et al. 2016) and colorectal (Du et al. 2008) cancers. In accordance with our results, various studies have demonstrated an increased uptake of HA‐decorated EVs by CD44‐expressing cells (Babula et al. 2023; Kim et al. 2025; Li et al. 2020; Lim et al. 2021; Liu et al. 2019; Liu et al. 2024; Soares et al. 2025; Yu et al. 2024). However, few studies have focused on the role of CD44 in the binding and uptake of the HA‐decorated EVs (Soares et al. 2025). Most in vitro studies compare nondecorated and HA‐decorated EVs in a single CD44‐expressing cell line (Babula et al. 2023; Kim et al. 2025; Liu et al. 2019; Liu et al. 2024; Yu et al. 2024), which does not exclude the role of other possible receptors for EV uptake. Alternatively, two or more different cell lines with varying CD44 expressions were used to compare HA‐decorated EV uptake (Li et al. 2020; Soares et al. 2025), which in turn might overlook cell‐type‐specific uptake mechanisms. To address these limitations, we utilised fully CD44‐deficient and CD44‐expressing variants of a single cell line as target cells to specifically assess the impact of CD44 on EV uptake.

To examine the role of CD44 in uptake of HA‐decorated EVs, we used free HA to saturate the CD44 receptors. Unexpectedly, free HA reduced uptake of both non‐HA‐decorated MCF7‐EVs and HA‐decorated HAS3‐EVs in both CD44^+^ and CD44^−^ cells. Similar effects have been reported with HA‐engineered milk EVs (Soares et al. 2025). Other studies observed decreased uptake of HA‐decorated EVs due to addition of free HA, but uptake of nondecorated EVs has not been reported (Liu et al. 2019; Yu et al. 2024). In addition to CD44‐blocking, the reduced uptake may be a result of the high HA concentrations increasing medium viscosity or creating physical barriers. Antibody‐based CD44 blocking has shown variable effects across studies (Babula et al. 2023; Liu et al. 2024), suggesting that CD44 contributes partially to HA‐EV uptake. Depending on cell type, other receptors like TLR4 (Kim et al. 2025) or HARE (Harris and Baker 2020) may also mediate HA‐decorated EV binding. Interestingly, CD44‐expression level of cells does not always correlate with internalisation rate of HA. For example, CD44s promotes HA uptake in cancer cells but not fibroblasts, while CD44v may inhibit it (Spadea et al. 2019). In the present study, GFP‐HAS3‐EVs mainly localised as bound to the membrane of CD44^+^ cells, highlighting the complex role of CD44 in EV internalisation.

To assess endocytosis mechanisms of HA‐decorated EVs and the role of CD44 in EV internalisation, we inhibited common endocytosis pathways (Mulcahy et al. 2014) using chlorpromazine (clathrin‐mediated) and EIPA (macropinocytosis). Both inhibitors reduced uptake of both nondecorated and HAS3‐decorated EVs, independent of CD44 expression. Previous studies have shown that blocking clathrin‐mediated endocytosis reduced the uptake of HA‐functionalised milk‐EVs by MDA‐MB‐231 breast cancer cells more than nonfunctionalised EVs (Soares et al. 2025). In our experiments, CD44^+^ and CD44^−^ cells took up MCF7‐EVs at similar levels across treatments. However, CD44^+^ cells internalised more HAS3‐EVs than CD44^−^ cells when clathrin‐mediated endocytosis and macropinocytosis were inhibited. This suggests that CD44‐mediated uptake of HAS3‐EVs may rely less on these pathways. MβC treatment, which blocks lipid raft mediated endocytosis by depleting plasma membrane cholesterol, surprisingly increased the uptake of both types of labelled EVs into CD44^+^ and CD44^−^ cells. A similar effect was previously observed in astrocytes treated with brain‐derived EVs (Pantazopoulou et al. 2023), though other studies have reported reduced EV uptake at comparable or higher MβC concentrations (Escrevente et al. 2011; Koumangoye et al. 2011; Perez et al. 2022; Svensson et al. 2013). The observed increase in MKN74 cells could be a result of other endocytosis pathways overcompensating for a loss of cholesterol‐dependent pathways, as similar effect was not observed with breast epithelial and breast cancer cells. It should also be noted that a drastic change was observed in the morphology of MKN74 cells after cholesterol depletion. In literature, suggested mechanisms for EV uptake are conflicting (Verdera et al. 2017; Li et al. 2020), thus it is likely that EV uptake mechanisms vary depending on both the EV subtype and the recipient cell type.

In addition to CD44‐mediated targeting demonstrated in the present and previous studies, HA in the pericellular matrix has been suggested to help tether HA‐decorated EVs to CD44^+^ cells, acting like a sponge (Ragni et al. 2020, 2019). Supporting this, we found that hyaluronidase digestion of pericellular HA blocked binding of HA‐decorated EVs, despite CD44 expression in the cells. Similar effects have been reported in nanoparticle studies (Liu et al. 2024; Zhou et al. 2012). Regardless, the role of the ECM and especially glycocalyx in EV tethering and uptake remains elusive and could be addressed in a future study.

To assess the comprehensive role of HA‐decoration in the physicochemical properties of EVs, we examined its impact on the formation of protein corona – soluble proteins that bind or adsorb onto the surface of EVs. Although the definition of the corona is still evolving, the term biomolecular corona is currently used to describe all the acquired molecules on EV surface (Esmaeili et al. 2025). The spontaneous formation of protein corona around EVs in plasma has been studied previously (Dietz et al. 2023; Palviainen et al. 2020; Tóth et al. 2021), and it has gained increasing interest after demonstration of its impact on the uptake of EVs into immune cells (Dietz et al. 2023), enhancing regenerative functions (Wolf et al. 2022) and altering biodistribution (Liam‐Or et al. 2024).

We utilised CD9‐antibody mediated capture of EVs for MP‐SPR‐based plasma protein corona formation, which allowed us to investigate a subpopulation of EVs, that was expected to be mainly plasma membrane origin and be enriched with HA‐decorated EVs. Interestingly, HAS3‐EVs formed a high‐affinity protein corona nearly twice as thick as that of nondecorated EVs. Mass spectrometry confirmed this finding, revealing significant enrichment of known plasma corona proteins. Out of the nine universal corona proteins (APOA1, APOB, APOC3, APOE, C3, C4B, FGA, IGHG2 and IGHG4) suggested by Tóth et al. (2021), all except C4B were identified in both plasma‐exposed MCF7‐EVs and HAS3‐EVs. Additionally, C3, FGA, IGHG2 and IGHG4 were among the significantly enriched proteins in plasma‐exposed HAS3‐EVs, which further supported the findings of increased corona diameter from the SPR experiments.

The effect of HA‐decoration on protein corona formation has been previously studied only with synthetic nanoparticles. HA‐decoration of chitosan nanoparticles led to reduced protein adsorption from bovine serum compared to alginate‐decorated and nondecorated particles (Almalik et al. 2017). The corona composition of HA‐decorated particles included inter‐alpha‐trypsin inhibitor ITIH4 and Complement 3, which were also enriched in our plasma‐exposed HAS3‐EVs. Plasma‐derived protein corona formation of HA‐decorated liposomes using the same MP‐SPR method (Kari et al. 2020) previously resulted in corona thickness of 2–6 nm depending on liposome formulation, which is in line with the measured corona thickness for MCF7‐EVs and HAS3‐EVs (2.7 and 5.5 nm, respectively). Mass spectrometry analysis of liposomes indicated enrichment of fibrinogens, Complement 3 and immunoglobulin variable regions for all anionic formulations, and enrichment of fibronectin, and inter‐alpha‐trypsin inhibitors 2 and 4 in the HA‐decorated liposomes (Kari et al. 2020). Interestingly, aside from fibronectin, plasma‐exposed HAS3‐EVs in this study were significantly enriched with same proteins (FGA, C3 and ITIH4), suggesting that either increased anionic charge, which was around 1.5‐fold more negative for the HAS3‐EVs, or the presence of HA as such on the EV surface causes increased adsorption of these proteins.

Although recent studies have demonstrated corona‐dependent functions for EVs, the underlying mechanisms of biomolecular corona biogenesis and its functional role remain poorly understood. Corona formation and composition have been shown to differ based on culture conditions (Liam‐Or et al. 2024), EV isolation method (Wolf et al. 2022) and EV source (Musicò et al. 2024), even though some proteins are consistently identified. The heterogeneous subpopulations and intrinsic proteome of EVs also causes challenges for identification of acquired corona proteins. Methodology to reliably study corona formation is also currently limited, and the in vitro settings do not accurately represent the in vivo environment. For example, plasma must be EV‐depleted to study corona formation, which also removes lipoproteins known to interact with EVs (Busatto et al. 2020), potentially altering EV behaviour (Busatto et al. 2022). Therefore, drawing conclusions on the biological significance of the corona composition is challenging. In our study, incubation with EV‐depleted FBS or plasma increased the uptake of HAS3‐EVs into both CD44^+^ and CD44^−^ cells. Our results are in agreement with previous study where plasma incubation increased the uptake of colorectal carcinoma cell EVs into fibroblasts, monocyte‐derived dendritic cells and colorectal cancer cells (Dietz et al. 2023). Serum‐incubated mesenchymal stem cell EVs on the other hand showed either decrease or increase in uptake depending on cell type and donor cell culture conditions (Liam‐Or et al. 2024). It should be noted that in these studies the incubation was done in 50%–100% FBS or plasma and EVs were then pelleted using ultracentrifugation, in contrast to our 5% FBS/plasma incubation without further isolation. Additionally, lipophilic dye exchange from EVs to lipoproteins or other plasma components may account into the observed increased uptake (Brealey et al. 2024).

We found that the HAS3‐EVs were enriched with immunomodulatory proteins, namely immunoglobulin components and complement factors. That could indicate a rapid clearance of the HA‐decorated EVs by macrophages or dendritic cells in circulation. HA itself is nonimmunogenic and used as decoration in synthetic nanocarriers due to its anionic charge, which should prolong the circulation time of nanoparticles. In an in vivo study using click‐chemistry glycoengineered PEGylated HA‐EVs, enhanced targeting to inflamed joints compared to control EVs was observed in mice, along with colocalisation of fluorescent EV signal with CD44 in the synovium (Lim et al. 2021). In the same study, similar effect was observed in mice bearing PC3 prostate cancer tumours, and it was hypothesised that the effect of enhanced targeting capability of HA‐EVs may result from their prolonged circulation time. In a recent follow‐up study, PEGylated HA‐EVs were compared with HA‐conjugated EVs without PEG, and with EVs without glycoengineering in a mouse acute kidney injury model (Kim et al. 2025). A similar plasma clearance was observed among EVs, but PEGylated HA‐EVs and especially HA‐conjugated EVs without PEG accumulated more in the liver and kidneys, colocalising with CD44‐expressing cells. Similarly, lipid‐grafted HA‐EVs loaded with doxorubicin showed improved tumour targeting and therapeutic efficacy without increased liver accumulation (Liu et al. 2019). These data indicate that despite no apparent benefit from HA‐decoration in circulation time or escape from liver‐mediated EV clearance, HA‐decorated EVs have targeting potential into CD44‐expressing cells. Furthermore, the influence of the protein corona on EV–target cell interactions, and its potential role in HA–CD44 binding, is an intriguing topic that warrants more detailed investigation.

Elucidating the EV surface functionality and its effects on biomolecular corona formation can provide new insight on the long‐standing questions regarding the biological processes driving EV targeting and cellular uptake. Moreover, understanding the carbohydrate structures on EVs can pave the way for novel glycoengineering strategies, which have already shown potential in EV targeting in vivo (Kim et al. 2025; Zheng et al. 2022).

## Funding

This project was funded by the Research Council of Finland Flagship Project GeneCellNano (K.R.), the Doctoral Programme in Molecular Medicine at University of Eastern Finland (H.K.) and by personal grants (H.K.) from The Savo Cancer Fund and Finnish Cultural Foundation. Partial funding was received from Finnish Research Impact Foundation (M.H., E.S. and T.V.), the Doctoral Programme in Materials Research and Nanoscience at University of Helsinki (E.S.) and Business Finland Data‐Inductor project (3898/31/2024) (M.H., E.S. and T.V.).

## Conflicts of Interest

The authors declare no conflicts of interest.

## Permission to Reproduce Material From Other Sources

Proteomics data used in Figure 7E were obtained from articles licensed under the Creative Commons Attribution 4.0 International License (CC BY 4.0). These data were reused in accordance with the license terms, and appropriate attribution has been provided.

## Supporting information




**Supporting Information**: jev270263‐supp‐0001‐Table_S1

**Supporting Information**: jev270263‐supp‐0002‐Table_S2

**Supporting Information**: jev270263‐supp‐0003‐Table_S3

**Supporting Information**: jev270263‐supp‐0004‐Table_S4

**Supporting Information**: jev27063‐sup‐0002‐SuppMat.pdf

**Supporting Information**: jev27063‐sup‐0001‐SuppMat.docx

## Data Availability

All data supporting the results of this study are available within the article and its Supporting Information. Any additional information is available from the corresponding author upon reasonable request. Mass spectrometry data have been uploaded to the MassIVE repository with accession code MSV000099330.
