# Supplementary material for: Hyaluronic Acid Decoration Facilitates CD44‐Mediated Targeting and Alters Protein Corona Formation of Extracellular Vesicles
Source: J Extracell Vesicles. 2026 Mar 26;15(4):e70263. doi: 10.1002/jev2.70263 (PMC13140515; doi:10.1002/jev2.70263)
Supplement: Supplementary file 5 — Supporting Information: jev27063‐sup‐0002‐SuppMat.pdf [file JEV2-15-e70263-s002.pdf]

Analysis Sequence "3D_spots_analysis_MKN74_PKH26_EV_uptake_2.0"

Input Image	Input
	**Channel group :** 1<br>**Sequences :** ALL<br>**Flatfield Correction :** None<br>Brightfield Correction<br>**Stack Processing :** 3D Analysis

Find Image Region	Input	Method	Output
	**Channel :** Alexa 647 short<br>**ROI :** None	**Method :** Absolute Threshold<br>Lowest Intensity : ≥ 300<br>Highest Intensity : ≤ INF<br>Filling : Fill Plane-Wise<br>Join touching Fragments<br>Volume : > 1000 µm³	Output Population : CD44 area<br>Output Region : CD44

Find Image Region (2)	Input	Method	Output
	**Channel :** Alexa 647 short<br>**ROI :** None	**Method :** Absolute Threshold<br>Lowest Intensity : ≥ 0<br>Highest Intensity : ≤ 300<br>Join touching Fragments<br>Volume : > 1000 µm³	Output Population : MOCK area<br>Output Region : MOCK

Find Nuclei	Input	Method	Output
	**Channel :** HOECHST 33342<br>**ROI :** CD44 area<br>**ROI Region :** CD44	**Method :** B<br>Common Threshold : 0.4<br>Volume : > 120 µm³<br>Splitting Coefficient : 7<br>Individual Threshold : 0.4<br>Contrast : > 0.1<br>Accuracy / Speed : Standard / Standard	Output Population : CD44 nuclei

Find Nuclei (2)	Input	Method	Output
	**Channel :** HOECHST 33342<br>**ROI :** MOCK area<br>**ROI Region :** MOCK	**Method :** B<br>Common Threshold : 0.4<br>Volume : > 120 μm³<br>Splitting Coefficient : 7<br>Individual Threshold : 0.4<br>Contrast : > 0.1<br>Accuracy / Speed : Standard / Standard	Output Population : MOCK nuclei

Find Cytoplasm	Input	Method	Output
	**Channel :** EGFP<br>**Nuclei :** CD44 nuclei	**Method :** A<br>Individual Threshold : 0<br>Restrictive Region : CD44<br>Accuracy / Speed : Standard / Standard	

Find Cytoplasm (2)	Input	Method	Output
	**Channel :** EGFP<br>**Nuclei :** MOCK nuclei	**Method :** A<br>Individual Threshold : 0.04<br>Restrictive Region : MOCK<br>Accuracy / Speed : Standard / Standard	

Filter Image	Input	Method	Output
	**Channel :** PKH26	**Method :** Sliding Parabola<br>Curvature : 100	Output Image : Sliding Parabola

Find Image Region (3)	Input	Method	Output
	**Channel :** Sliding Parabola<br>**ROI :** CD44 nuclei<br>**ROI Region :** Cell	**Method :** Absolute Threshold<br>Lowest Intensity : ≥ 10<br>Highest Intensity : ≤ INF<br>Join touching Fragments<br>Volume : > 0.2 μm³	Output Population : CD44 EV<br>Output Region : CD44 EV region

Find Image Region (4)	Input	Method	Output

	**Channel :** Sliding Parabola<br>**ROI :** MOCK nuclei<br>**ROI Region :** Cell	**Method :** Absolute Threshold<br>Lowest Intensity : ≥ 10<br>Highest Intensity : ≤ INF<br>Join touching Fragments<br>Volume : > 0.2 μm³	Output Population : MOCK EV<br>Output Region : MOCK EV region

**Calculate Properties**	**Input**	**Method**	**Output**
	**Population :** CD44 nuclei	**Method :** By Related Population<br>Related Population : CD44 EV<br>Number of CD44 EV	Property Suffix : per Cell

**Calculate Properties (2)**	**Input**	**Method**	**Output**
	**Population :** MOCK nuclei	**Method :** By Related Population<br>Related Population : MOCK EV<br>Number of MOCK EV	Property Suffix : EV per MOCK cell

**Calculate Morphology Properties**	**Input**	**Method**	**Output**
	**Population :** CD44 EV<br>**Region :** CD44 EV region	**Method :** Standard<br>Volume	Property Prefix : CD44 EV volume

**Calculate Morphology Properties (2)**	**Input**	**Method**	**Output**
	**Population :** MOCK EV<br>**Region :** MOCK EV region	**Method :** Standard<br>Volume	Property Prefix : MOCK EV volume

**Calculate Morphology Properties (3)**	**Input**	**Method**	**Output**
	**Population :** CD44 nuclei<br>**Region :** Cell	**Method :** Standard<br>Volume	Property Prefix : CD44 volume

**Calculate Morphology Properties (4)**	**Input**	**Method**	**Output**

	**Population :** MOCK nuclei<br>**Region :** Cell	**Method :** Standard Volume	Property Prefix : MOCK volume

Define Results	Results
	**Method :** List of Outputs<br>**Population : CD44 EV**<br>Number of Objects<br>CD44 EV volume Volume [µm³] : Sum<br><br>**Population : MOCK EV**<br>Number of Objects<br>MOCK EV volume Volume [µm³] : Sum<br><br>**Population : CD44 nuclei**<br>Number of Objects<br>Number of CD44 EV- per Cell : Mean<br>CD44 volume Volume [µm³] : Sum<br><br>**Population : MOCK nuclei**<br>Number of Objects<br>Number of MOCK EV- EV per MOCK cell : Mean<br>MOCK volume Volume [µm³] : Sum<br><br>**Method :** Formula Output<br>Formula : a/b<br>Population Type : Objects<br>Variable a : CD44 EV - CD44 EV volume Volume [µm³] Sum<br>Variable b : CD44 nuclei - CD44 volume Volume [µm³] Sum<br>Output Name : CD44 EV/cell volume<br><br>**Method :** Formula Output<br>Formula : a/b<br>Population Type : Objects<br>Variable a : MOCK EV - MOCK EV volume Volume [µm³] Sum<br>Variable b : MOCK nuclei - MOCK volume Volume [µm³] Sum<br>Output Name : MOCK EV/cell volume<br><br>**Object Results**<br>Population : CD44 area : None<br>Population : MOCK area : None<br>Population : CD44 EV : None<br>Population : MOCK EV : None<br>Population : CD44 nuclei : None<br>Population : MOCK nuclei : None

Acapella version: 5.6.1.132829. Timestamp: 2025-07-24 15:53:02 +0300.
