# Supplementary material for: Hyaluronic Acid Decoration Facilitates CD44‐Mediated Targeting and Alters Protein Corona Formation of Extracellular Vesicles
Source: J Extracell Vesicles. 2026 Mar 26;15(4):e70263. doi: 10.1002/jev2.70263 (PMC13140515; doi:10.1002/jev2.70263)
Supplement: Supplementary file 6 — Supporting Information: jev27063‐sup‐0001‐SuppMat.docx [file JEV2-15-e70263-s006.docx]

**Supplementary material**

**Supplementary materials and methods**

**Confocal imaging of MCF7-GFP-HAS3 cells**

MCF7-GFP-HAS3 cells (20 000 / well) were seeded on Ibitreat 8-well chamber glass (Ibidi GmbH, Gräfelfing, Germany) and incubated for 24 h. For induction of GFP-HAS3, 1 µg/ml doxcycycline (Sigma-Aldrich) was added and the cells were incubated for 24 h. To visualize the pericellular HA-coat, 0.25 µg/ml Alexa fluor 568-conjugated HABC (Rilla et al., 2008) was added. For visualisation of EV-marker expression, 1:200 FITC anti-human CD9 (#312103, Biolegend, San Diego, CA, USA), 1:200 PE anti-human CD9 (#312105, Biolegend), 1:200 Alexa fluor 488 anti-human CD63 (#353037, Biolegend), or 1:200 Alexa fluor 594 CD63 (#353033, Biolegend) were used. Cells were imaged live using Zeiss Axio Observer microscope (Carl Zeiss Microscopy GmbH), equipped with LSM800 confocal module (Carl Zeiss Microscopy GmbH) and 63 x NA 1.4 oil objective (Carl Zeiss Microscopy GmbH) using Zen Blue 2.3 software.

**HA-oligomer and hyaluronidase treatments**

MKN74 CD4 and MKN74 MOCK co-cultures (1:1) were prepared by seeding 20 000 cells per well on an IbiTreat 8-well chamber glass (Ibidi). After 24 h incubation, to block the HAS3-EV interactions with CD44, cells were pretreated with 0.5 mg/ml HA6 or HA14 oligomers (Seikagaku corporation, Tokyo, Japan), or with 0.1 mg/ml 2MDa HA (Lifecore Biomedical, Chaska, MN, USA) for 1 h before adding of 1x10^10^ GFP-HAS3 EVs. The cells were incubated with EVs for 6h before fixation.

For digestion of HA, 1 U/ml *Streptomyces* hyaluronidase (Sigma-Aldrich) was added to cell culture medium followed by incubation for 10 minutes at + 37 °C. Cells were then either washed twice with 1 X PBS before addition of EVs, or the EVs were added directly in the presence of the hyaluronidase. For digestion of HA-decoration on EVs, 1 U/ml *Streptomyces* hyaluronidase was added to the EV samples, followed by 10 min incubation at + 37 °C.

To visualize the pericellular HA-coat in MKN74 co-cultures, 0.25 µg/ml Alexa fluor 568-conjugated HABC (Rilla et al., 2008) was added to the cultures, followed by incubation of 30 minutes. CD44 expression was visualized by Alexa fluor 488 anti-human CD44 antibody (#397507, Biolegend) and nuclei were stained using NucBlue Fixed Cell ReadyProbes Reagent (Invitrogen), according to the manufacturer’s instructions. The cells were imaged live using Zeiss Axio Observer microscope (Carl Zeiss Microscopy GmbH), equipped with LSM800 confocal module (Carl Zeiss Microscopy GmbH) and 63 x NA 1.4 oil objective (Carl Zeiss Microscopy GmbH) using Zen Blue 2.3 software.

**HAS3-EV uptake into MCF10A and MCF10CA cells**

MCF10A breast epithelial and MCF10CA breast carcinoma cells were cultured in DMEM/F12 medium (Gibco, Thermo Fischer Scientific) supplemented with 5% horse serum (Invitrogen), 2 mM glutamine (EuroClone), 100 µg/mL streptomycin sulfate, 100 U/mL penicillin (EuroClone), 0.5 µg/mL epidermal growth factor (Sigma-Aldrich), 0.5 µg/mL hydrocortisone (Sigma-Aldrich), 0.1 µg/mL cholera toxin (Sigma-Aldrich), and 10 µg/mL insulin (Sigma-Aldrich). The cells were passaged twice a week with split ratios of 1:25 for MCF10A and 1:20 for MCF10CA using 0.05 % trypsin (w/v) 0.02 % EDTA (w/v) (Gibco). During experiments with EV-treatments, EV-depleted horse serum was used. EV-depleted horse serum was prepared by ultracentrifugation at 189 000 x *g* for 16 h, at + 4 °C, followed by sterile filtration with 0.22 µm pore size syringe filter (Sartorius).

For HAS3-EV uptake experiments, 15 000 MCF10A or MCF10CA cells were added per well on a PhenoPlate 96-well microplate (Revvity). After 24 h, 6x10^9^ PKH26-labelled HAS3-EVs were added to the culture, followed by 5h incubation before imaging. For endocytosis inhibition using methyl-β-cyclodextrin (MβC), 5 mM MβC was added to the cells 1h before addition of the PKH26-labelled HAS3-EVs. Before imaging, the cells were stained with Alexa fluor 488 anti-human CD44 antibody (#397507, Biolegend) and the nuclei were stained using NucBlue. The cells were then imaged live using Opera Phenix Plus high-content imaging system, equipped with 40 x water immersion objective (Revvity).

**MP-SPR Layer parameters**

To match the experimental spectra with the simulated ones in order to determine the biomolecular corona thickness for MCF7 EVs and HAS-3 EVs in LayerSolver software, the initial parameters of thickness (nm) and refractive index at 670 nm and 785 nm wavelengths are illustrated in table S1.

**Table S1. Starting optical parameters inserted in LayerSolver.**

| **Layer** | **Thickness (nm)** | **Real part at 670nm (*n*)** | **Imaginary part at 670 nm** (*k*) | **Real part at 785 nm** (*n*) | **Imaginary part at 785 nm** (*k*) |
| --- | --- | --- | --- | --- | --- |
| Glass | 0.00 | 1.52052 | 0.00 | 1.51793 | 0.00 |
| Cromium | 5.00 | 1.20541 | 3.35692 | 0.99572 | 0.05929 |
| Gold | 44.96 | 0.20610 | 3.81035 | 0.19692 | 4.83590 |
| Avidin | 4.05 | 1.38996 | 0.00 | 1.38815 | 0.00 |
| PBS | 0.00 | 1.33475 | 0.00 | 1.33301 | 0.00 |

**NanoLC-MS analysis of the EV protein content**

The eluates from the SPR experiments containing EVs and the biomolecular corona were collected, processed, and analysed with nanoflow liquid chromatography-mass spectrometry (nLC-MS).

For protein extraction, 100 µL of sample were adjusted to a pH of 8 using 1M NaOH (Sigma Aldrich, St. Louis, USA), and 75 mg of urea (Sigma Aldrich, St. Louis, USA) was added to the samples. Then, the samples were vortexed and sonicated for 15 min and finally centrifuged for another 15 minutes at 14,800 × g. After these procedures the supernatants were collected and processed for protein reduction and carbamidomethylation: dithiothreitol (DTT) (Sigma Aldrich, St. Louis, USA) was firstly added to the samples to the final concentration of 10 mM, followed by incubation at room temperature for 1 hour. Secondly, iodoacetamide (IAA) (Sigma Aldrich St. Louis, USA) was added to the final concentration of 50 mM and further incubated at room temperature for one hour. Eventually, to quench the carbamidomethylation reaction, additional DTT at the final concentration of 20 mM was added to the samples before protein precipitation.

In order to remove the detergents residues after collecting EVs from SPR, as well as excess DTT and IAA, protein precipitation was carried out with chloroform/methanol/water (4 volumes / 1 volume / 3 volumes) extraction to let the protein layer deposit at the interface between the aqueous and the organic solvents. At the end of the procedure the aqueous phase was removed, and 1 volume of methanol was further added. The samples were then mixed and centrifuged for 10 minutes at 14,800 × g to pellet the proteins. Successively, the pellets were resolubilized with 25 µL of a solution of 8M urea and 100 mM TEAB (Sigma Aldrich, St. Louis, USA), vortexed and sonicated for 15 minutes.

The total protein content of the samples was determined using Pierce BCA Protein Assay Kit (ThermoFischer,Rockford, IL, USA), and thereafter the samples were diluted with 100 mM TEAB to get 1.5M urea concentration. To normalize the samples based on total protein content, 4.64 µg of protein were taken from all samples for subsequent steps. Sequencing grade modified trypsin (PROMEGA, Madison, WI, USA) was dissolved in 100 mM TEAB at pH ~8 and added to proteins in a 1:50 enzyme-to-protein ratio and incubated overnight at 37°C with shaking.

Protein labelling was performed by adding tandem mass tags (TMTs) to the peptide solutions: TMT10plex Kit 126N TMT dry reagents and (ThermoFischer, Germany), were dissolved in 88µL of anhydrous acetonitrile (ACN), and then the solutions were added to each sample in order to get 20% v/v as final ACN concentration of and a 10:1 TMT:protein ratio. The samples were successively incubated for 1 hour at room temperature, followed by quenching the labeling reaction by adding 2.5 µL of 1% hydroxylamine (Sigma Aldrich). Eventually, the samples were incubated for 15 minutes at room temperature, combined into one Eppendorf tube, and dried for at least 2 hours in a vacuum centrifuge to evaporate most of the acetonitrile in them.

The samples were acidified to pH ~2 with 10% formic acid, followed by purification using C18 solid phase extraction (SPE) centrifuge cartridges (BioPureSPN Mini Proto 300 C18, 7–70 µg capacity, The Nest Group, Inc.). The SPE cartridges were conditioned with 2 × 200 µL of acetonitrile and equilibrated with 2 × 200 µL of 2% acetonitrile with 0.1% formic acid. The samples were loaded onto the cartridges and washed with 200 µL of 2% acetonitrile with 0.1% formic acid. Elution was performed first with 200 µL of 50% acetonitrile with 0.1% formic acid, followed by 200 µL of 80% acetonitrile with 0.1% formic acid. The eluted samples were evaporated to dryness using a vacuum centrifuge.

To increase the proteome coverage of the analysis, the samples were fractionated with C18 solid phase extraction (SPE) centrifuge cartridges. The multiplexed samples were dissolved in 2% ACN and 20 mM ammonium hydroxide (200 µl), vortexed and spun down and sonicated for 15 minutes. The fractionation was performed conditioning the cartridge with 200 µl of ACN twice, equilibrating it with 2% CAN and 20mM ammonium hydroxide twice, introducing the pooled samples and eluting them from the cartridge with 200 µl of 24 different eluents with an ACN concentration from 2% to 98%, in order to create 24 initial fractions, combined into 12 final fractions. Finally, the samples were evaporated to dryness using a vacuum centrifuge.

Prior to LC-MS analysis, the samples were reconstituted in 34 µL of eluent A (2% acetonitrile and 0.1% formic acid in water) by 15-minute sonication to get peptide concentration of 0.125 µg/µL and transferred into LC autosampler vials. LC-MS analysis was conducted with an EASY-nLC 1200 coupled to an Orbitrap Fusion mass spectrometer. Three microliters of the peptide solution were injected onto the C18 column system, consisting of a trap column (Thermo Acclaim PepMap™ 100, dimensions 75 µm × 2 cm, particle size 3 µm) and an analytical column (Thermo Acclaim PepMap™ RSLC, dimensions 75 µm × 15 cm, particle size 2 µm). The flow rate was 300 nL/min, and the gradient was as follows: 4 minutes with 5% eluent B, from 5% to 40% eluent B in 36 minutes, from 40% to 80% eluent B in 5 minutes, from 80% to 99% eluent B in 3 minutes. Then, the column was washed for 2 minutes with 99% eluent B, followed by decreasing the eluent B percentage to 1% in 1 min, and 5 minutes flush at 1% eluent B. Eluent A was 2% acetonitrile and 0.1% formic acid in water, and eluent B was 90% acetonitrile and 0.1% formic acid in water. The peptides were ionized in positive ionization mode with an electrospray voltage of 1800 V. Data-dependent acquisition with a cycle time of 3 seconds was used, in which precursors from each orbitrap MS1 full scan were fragmented and analyzed with MS2 in the orbitrap. EASY-IC internal mass calibration with fluoranthene was used at the start of the run. MS1 parameters were as follows: resolution 120000, scan range 375–1500 *m/z*, maximum injection time 50 ms, normalized AGC target 100%. MS2 parameters were as follows: quadrupole isolation window 1 *m/z*, HCD normalized collision energy 35%, resolution 50000, automatic scan range starting from 110 *m/z*, maximum injection time 150 ms, normalized AGC target 200%. Precursors were limited to peptides with monoisotopic precursor selection, charge states from 2 to 7, and intensities above 10,000. Precursor fit with 50% fit error and 1 *m/z* window was applied. A 60-second dynamic exclusion with 10 ppm mass tolerance was applied.

**Supplementary Data**


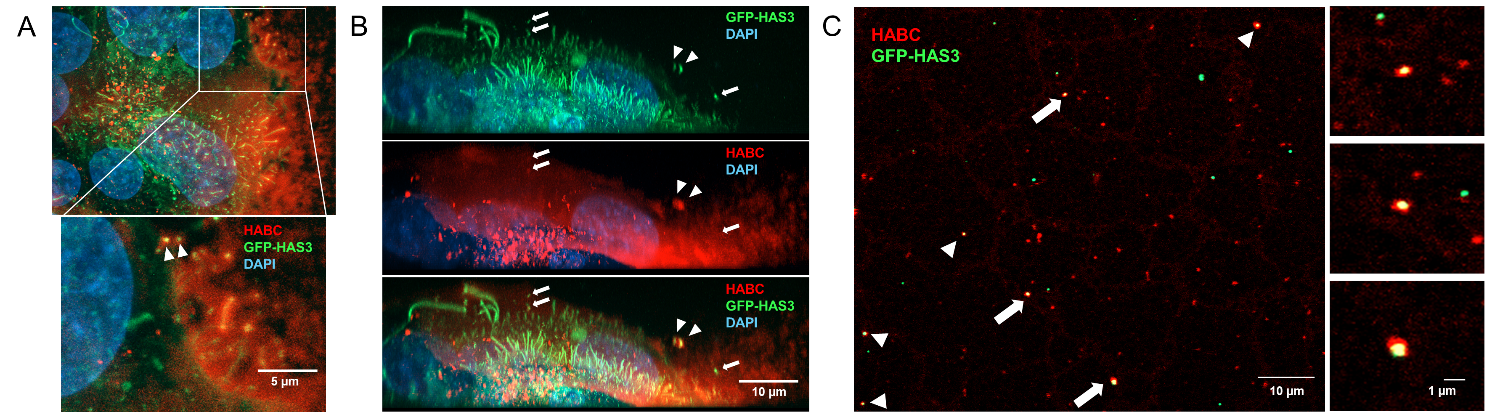
**Figure S1. Secretion of GFP-HAS3-EVs.** (A) Maximum intensity projection of GFP-HAS3-induced MCF7 cells secreting HA, visualised with HA-binding fluorescent probe (HABC). Area marked with a rectangle is magnified below and HA-decorated EVs are marked with arrowheads. (B) Side-view 3D projection of image A showing GFP-HAS3 and HA-positive EVs (arrows, arrowheads correspond with EVs in image A) secreted by cells. (C) Confocal image of ultracentrifugation-isolated GFP-HAS3-EVs. HA is visualised using the fluorescent HA-binding probe. Arrows point to EVs positive for both GFP-HAS3 and HA and magnified images of EVs pointed by arrowheads are shown on the right.


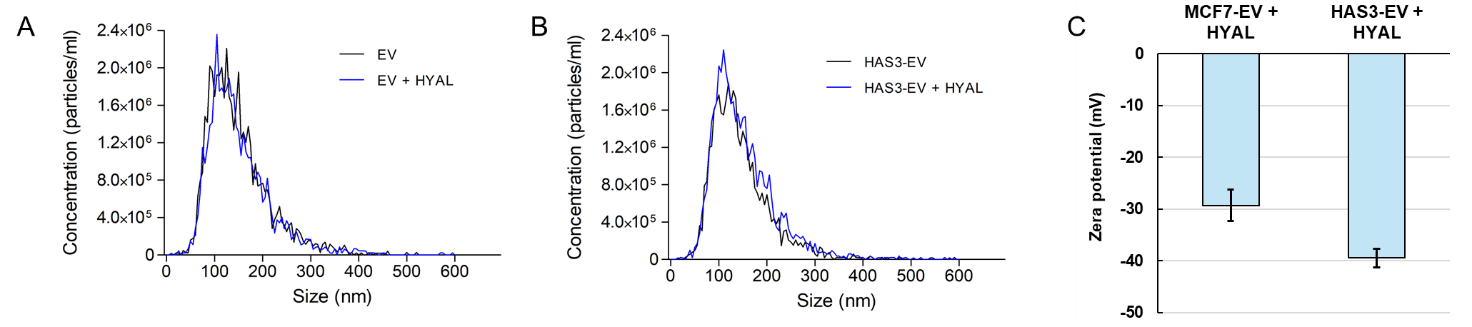

**Figure S2. Hyaluronidase treatment for MCF7-EVs and HAS3-EVs.** Nanoparticle tracking analysis size distribution for the effect of hyaluronidase treatment for (A) MCF7-EVs and (B) HAS3-EVs, n=3. (C) Zeta-potentials for hyaluronidase-treated MCF7-EVS and HAS3-EVs, n=3 with SEM.

**
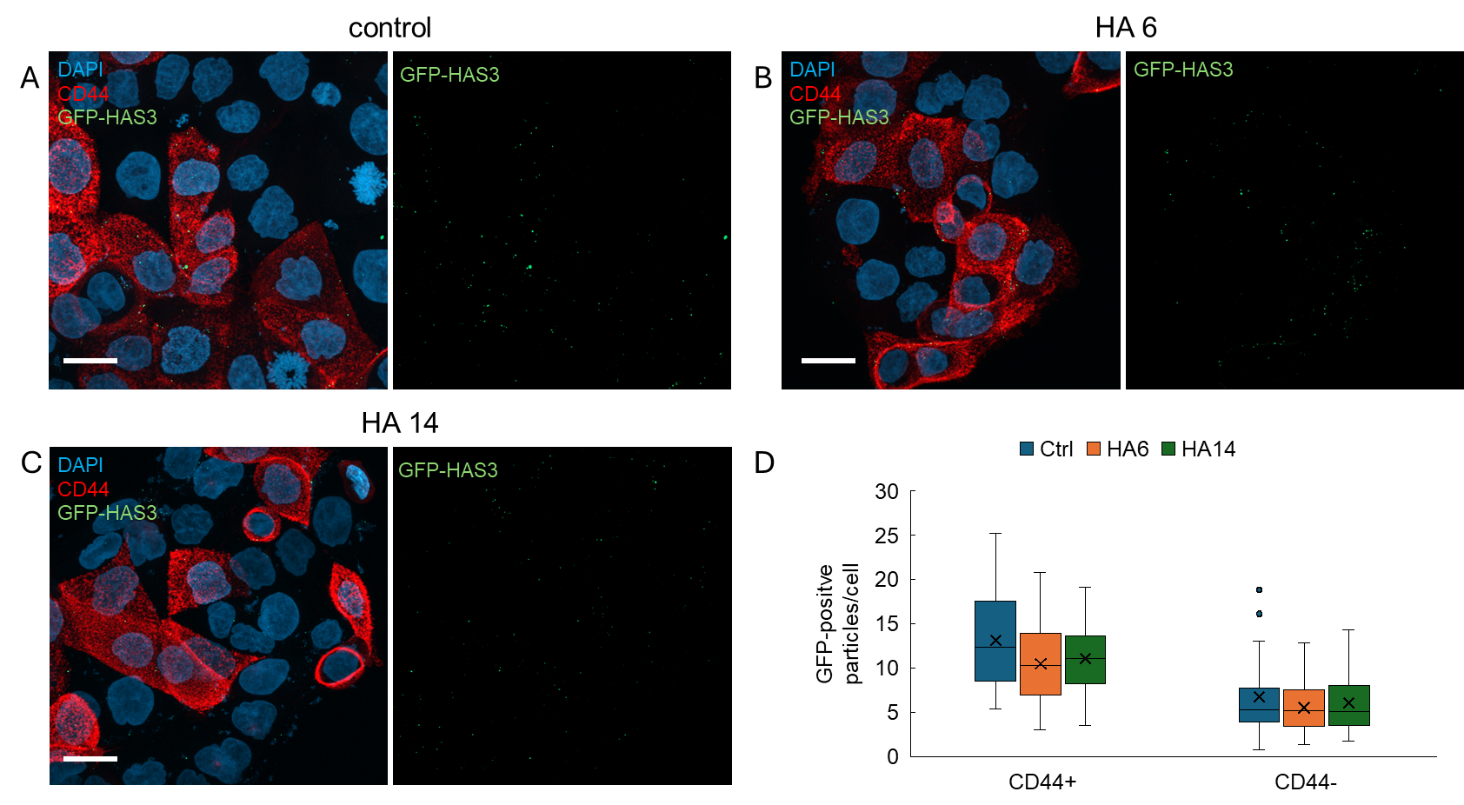
**
**Figure S3. The effect of HA-oligosaccharide treatment to GFP-HAS3-EV binding.** (A) Confocal microscopy image of fixed MKN74 co-culture after treatment with GFP-HAS3-EVs for 5h. The co-cultures were either treated with HA oligosaccharides of 6 sugar units (HA 6) (B) or 14 units (HA 14) (C). (D) The quantified GFP-positive particles in CD44+ and CD44- cells (n=5, a total of 298-388 CD44+ cells and 370-400 CD44- cells). Scale bars correspond to 20 µm.


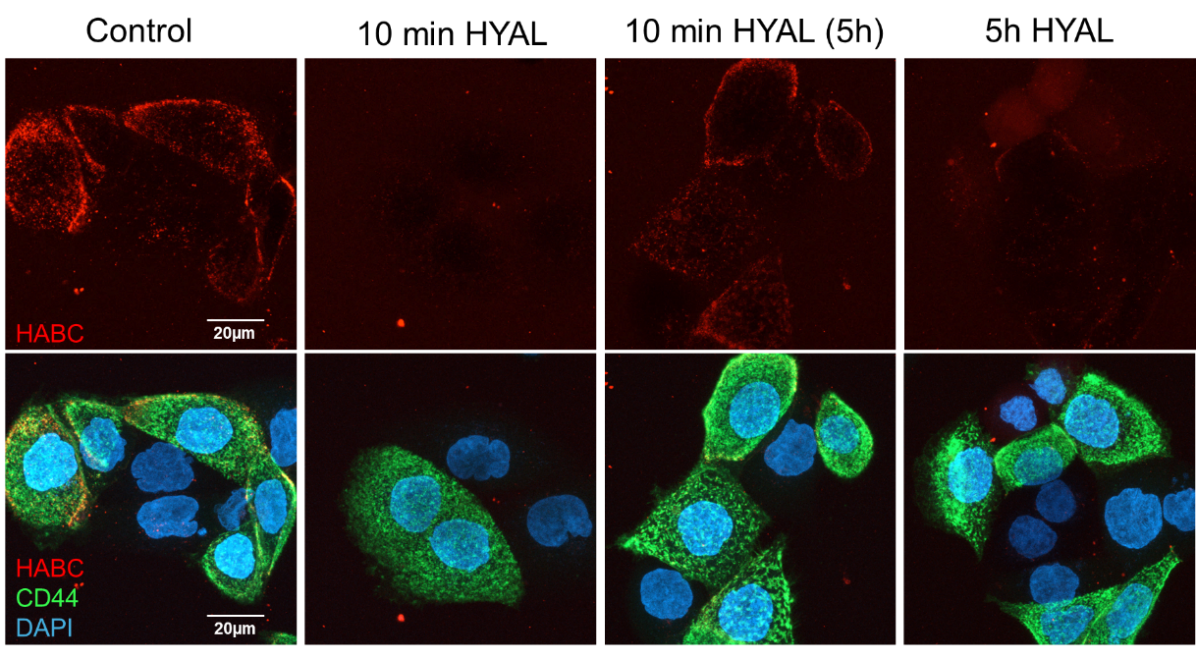

**Figure S4. HA-staining of hyaluronidase-treated MKN74 cultures.** Confocal microscopy images of live MKN74 co-cultures with HA visualised using fluorescent HA-binding probe (HABC). Images were taken from untreated cells (control), after 10 min hyaluronidase (HYAL) treatment (10 min HYAL), after 5h of the HYAL treatment (10 min HYAL (5h)), and cells after 5h with HYAL present the whole time.

**
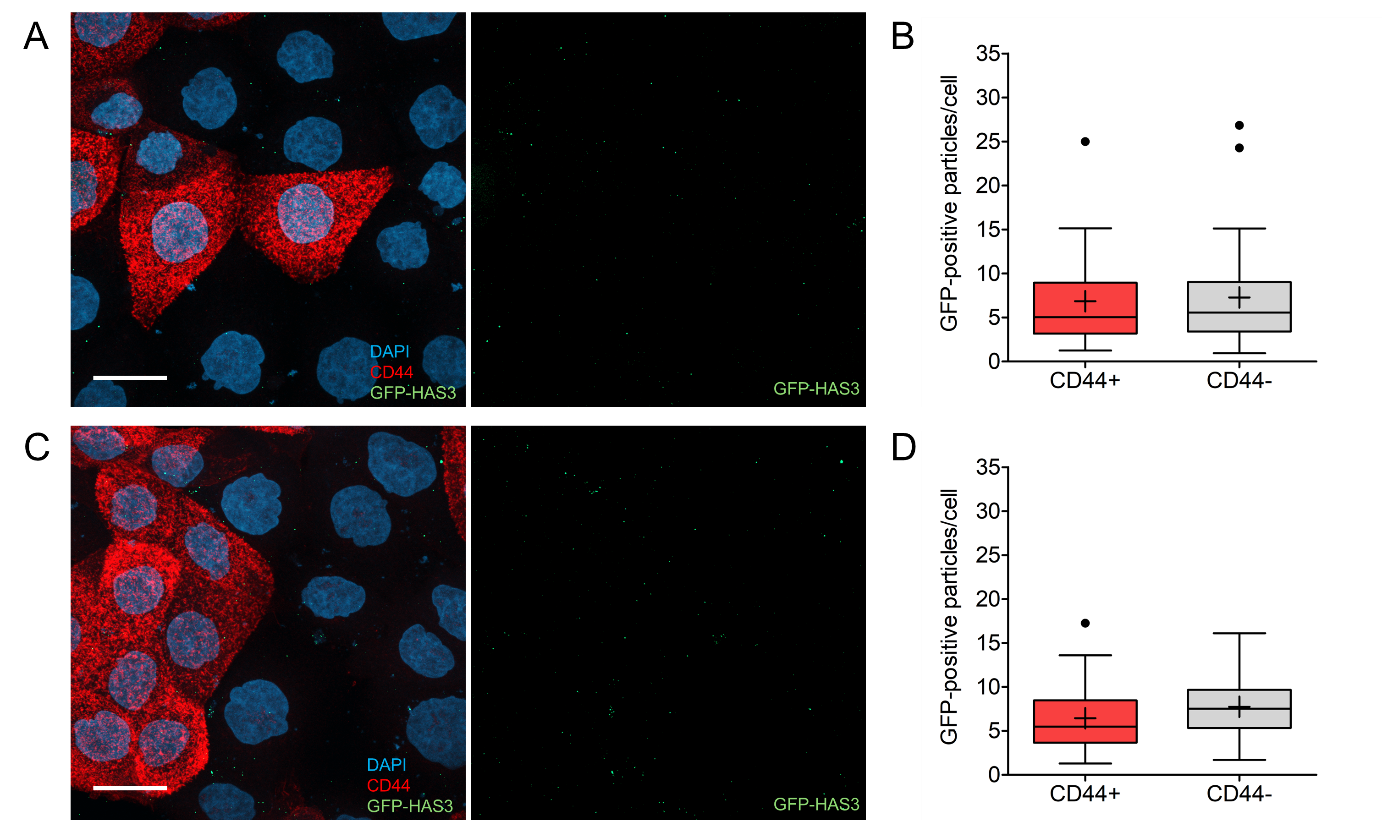
**
**Figure S5. The effect of hyaluronidase treatment on binding of GFP-HAS3-EVs.** Confocal microscopy images of fixed MKN74 co-cultures after 5h treatment with GFP-HAS3-EVs. Either the cell cultures before EV treatment (A) or EVs only before incubation with cells (C) were pretreated with hyaluronidase. (B, D) The number of GFP-positive particles was quantified from the images (n=4, cells pretreated 264 CD44+ and 345 CD44- cells, EVs pretreated 273 CD44+ and 358 CD44- cells). Scale bars correspond to 20 µm.

**
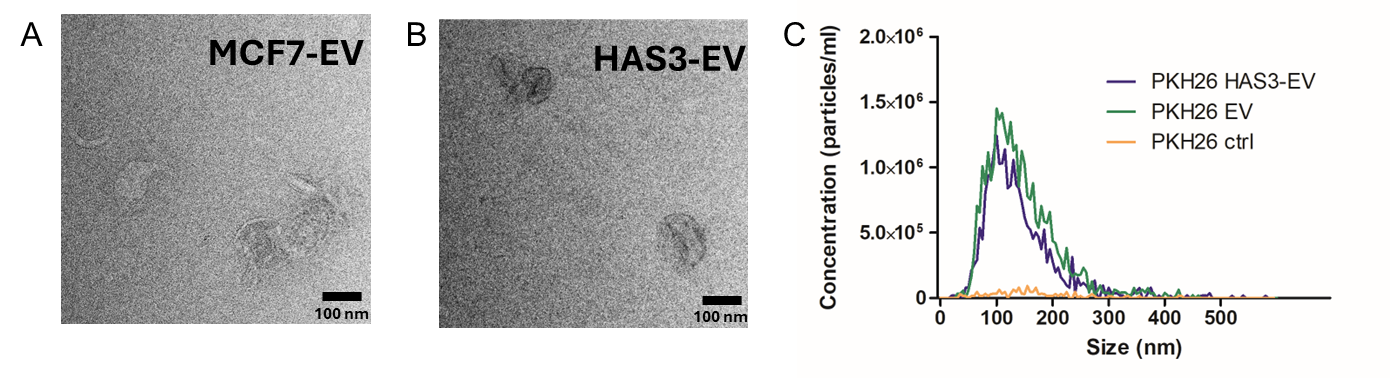
**
**Figure S6. Characterization of PKH26-labelled EVs.** Transmission electron microscopy images of MCF7-EVs (A) and HAS3-EVs (B) showing intact cup-shaped EVs after PKH26 staining. (C) Nanoparticle tracking analysis size-distribution of PKH26-stained MCF7-EVs, HAS3-EVs, and EV-free PKH26 dye control (n=3).

**
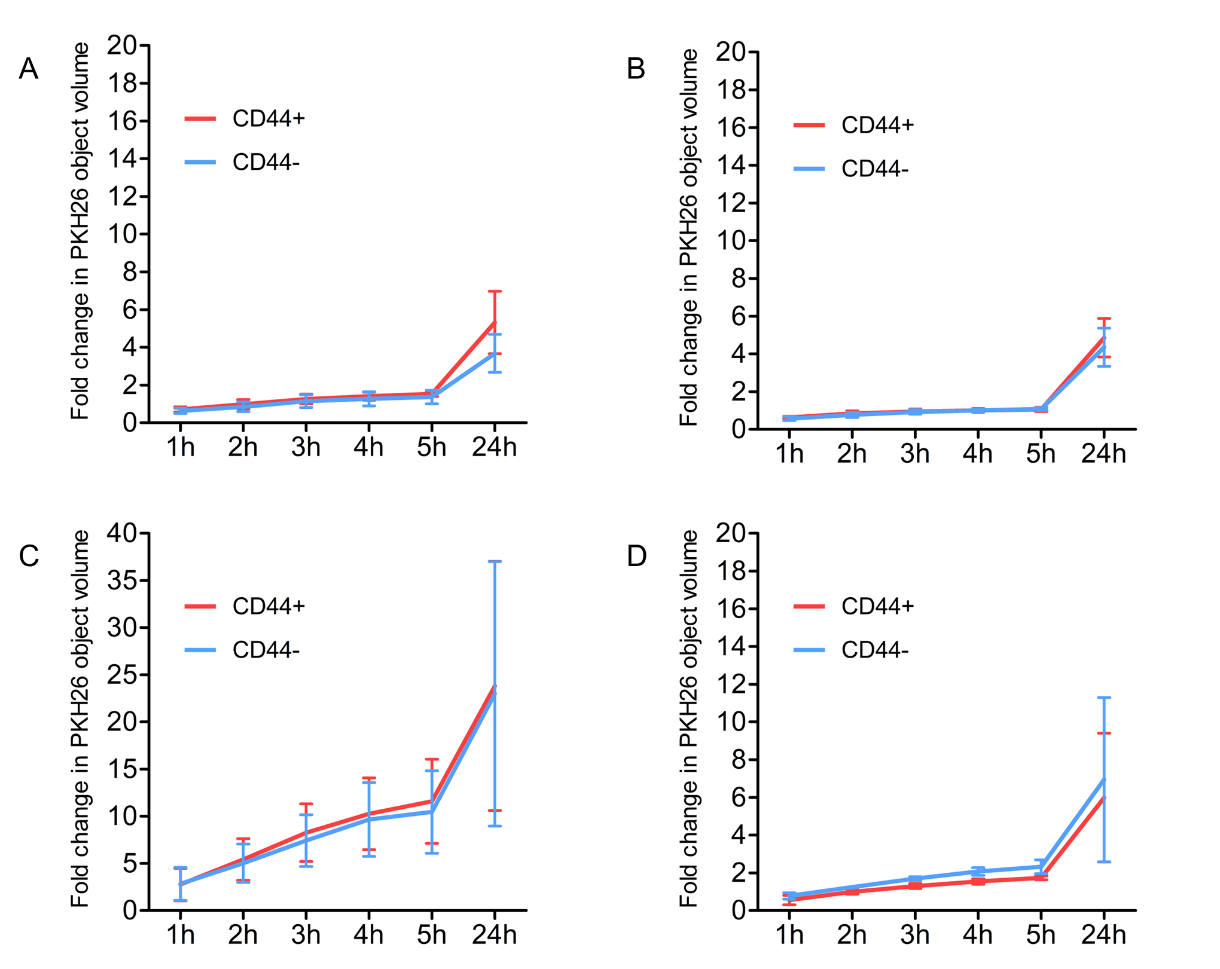
**
**Figure S7. The effect of endocytosis inhibitors on MCF7-EV uptake.** Time-based uptake of MCF7-EVs in MKN74 co-cultures in the presence of (A) chlorpromazine, (B) EIPA, (C) MβC, and (D) 2MDa HA.

**
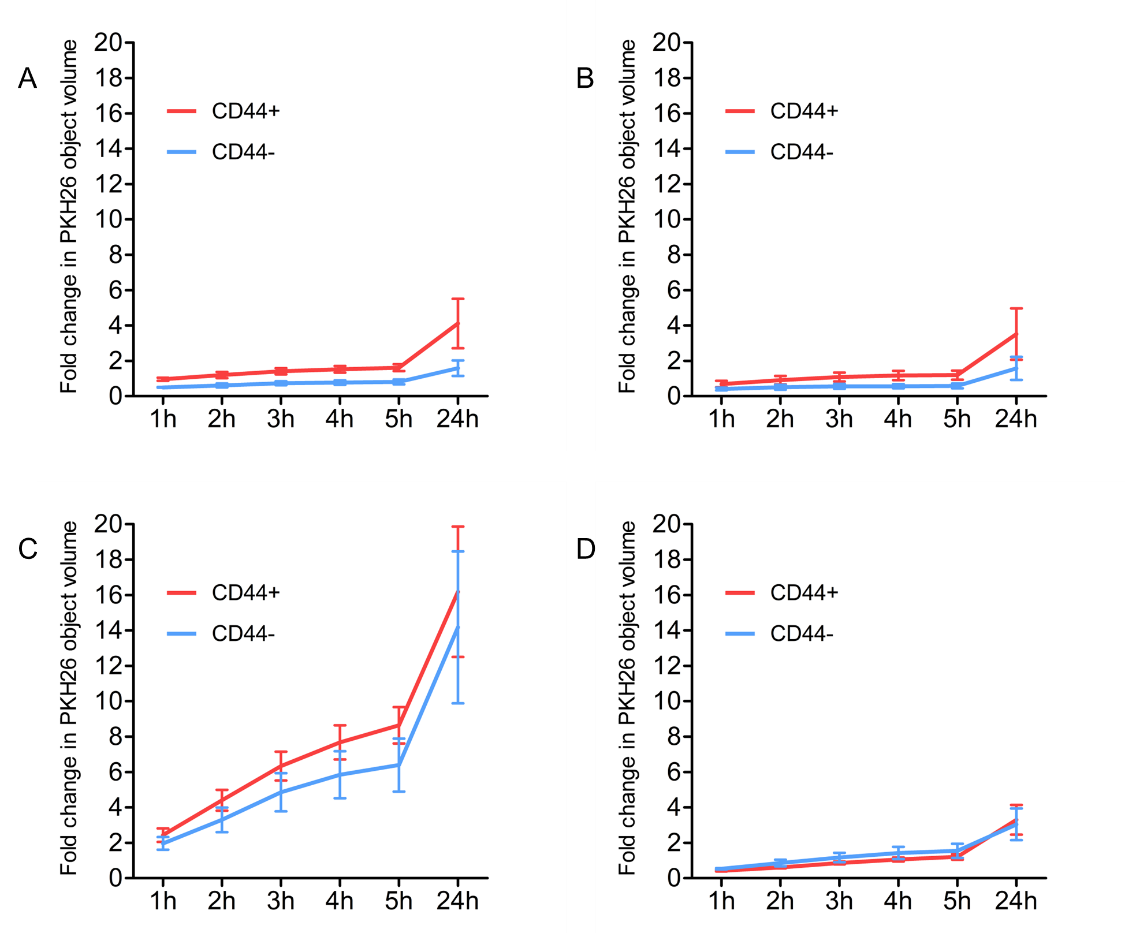
**
**Figure S8. The effect of endocytosis inhibitors on HAS3-EV uptake.** Time-based uptake of HAS3-EVs in MKN74 co-cultures in the presence of (A) chlorpromazine, (B) EIPA, (C) MβC, and (D) 2MDa HA.


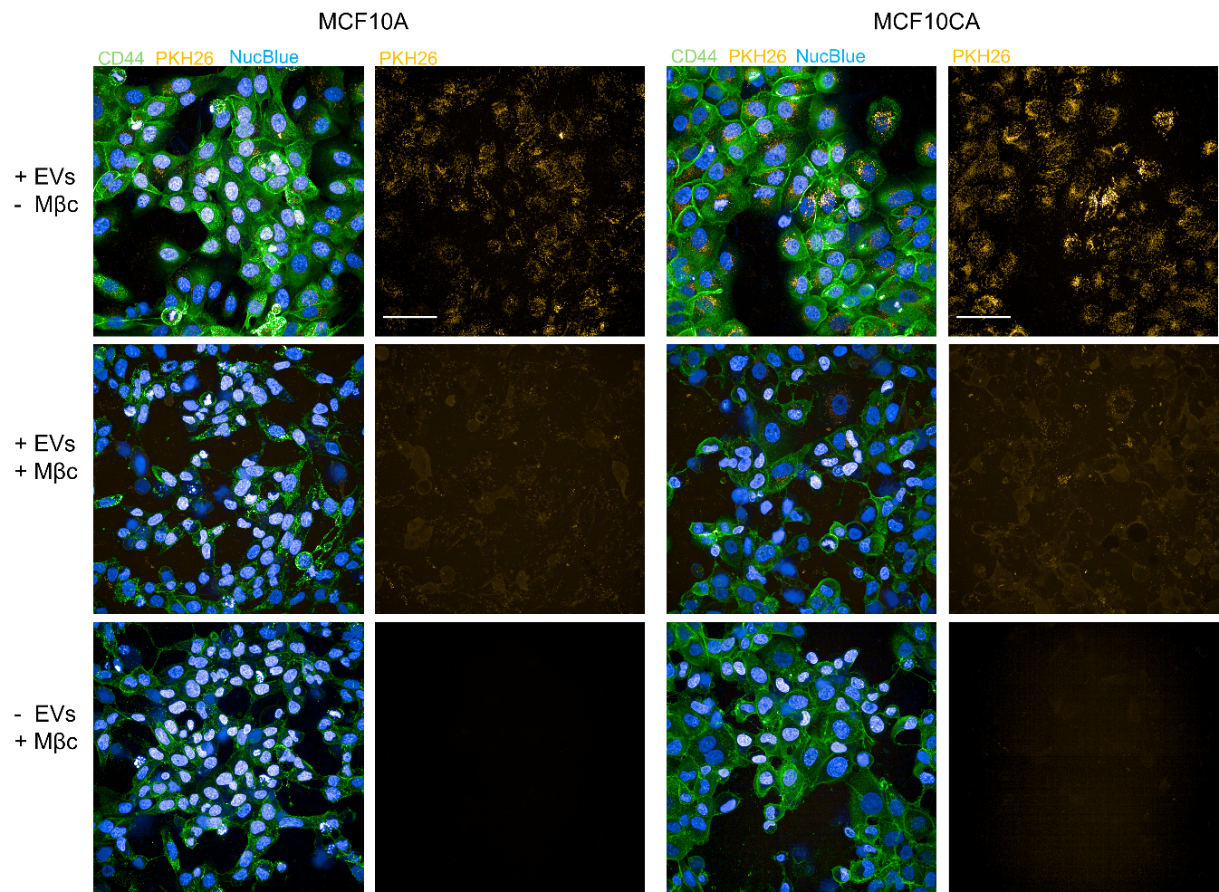

**Figure S9. The effect of methyl-β-cyclodextrin treatment on HAS3-EV uptake in MCF10A and MCF10CA cells.** MCF10A breast epithelial and MCF10CA breast cancer cells were treated with PKH26-labelled HAS3-EVs without, or with presence of MβC. Control MβC treatment without EVs confirmed the morphological change and lack of MβC-related autofluorescence. The scale bars represent a length of 50 µm.


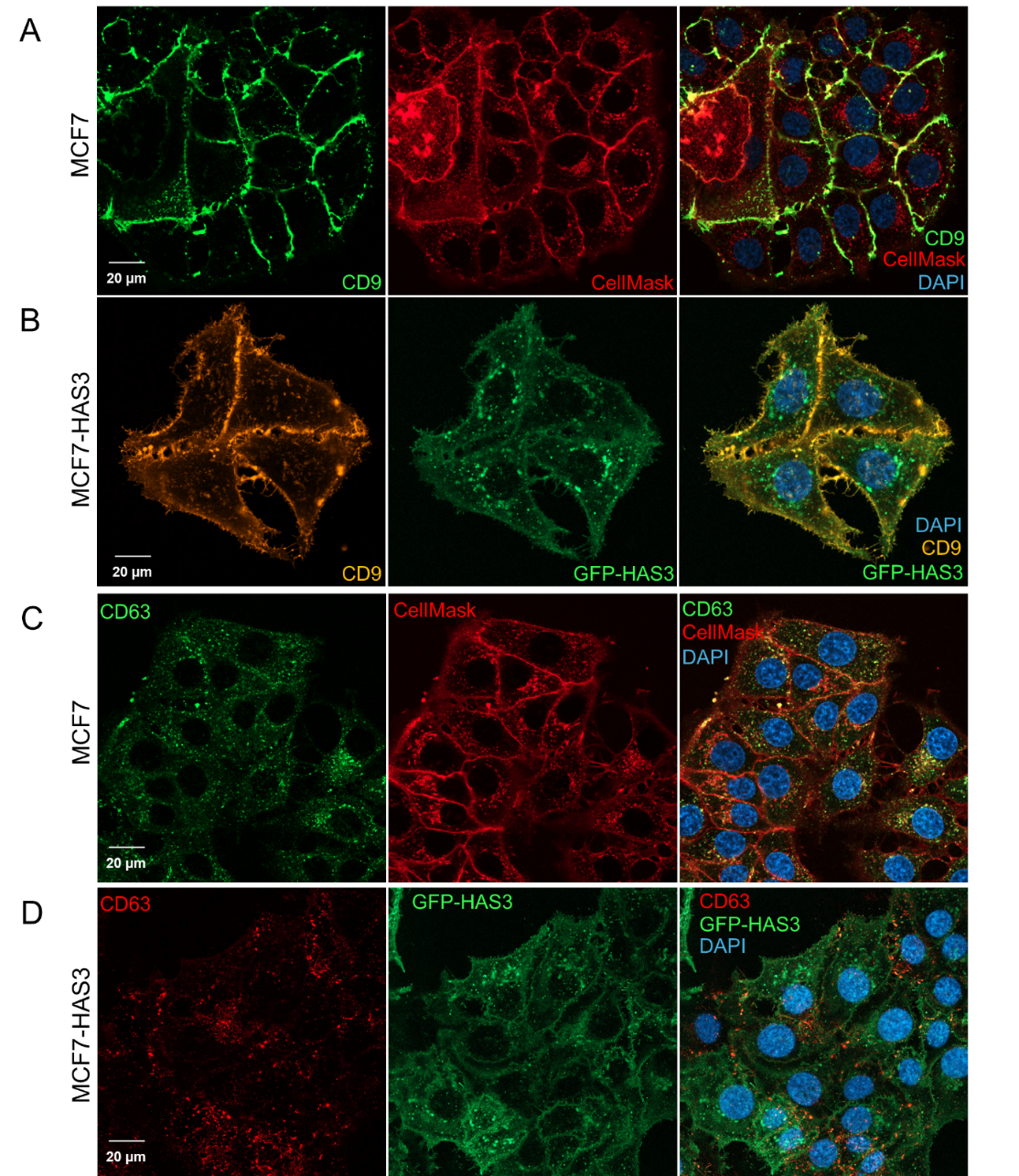

**Figure S10. Localization of CD9 and CD63 in MCF7 and MCF7-GFP-HAS3 cells.** Confocal images of live MCF7 cells and MCF7-GFP-HAS3 cells with fluorescent antibody labelled CD9 and CD63. CD9 primarily localizes on the plasma membrane in both MCF7-cells (A) and GFP-HAS3-induced MCF7 cells (B), while CD63 is primarily localized in the intracellular endosomal compartments in both cells (C-D).


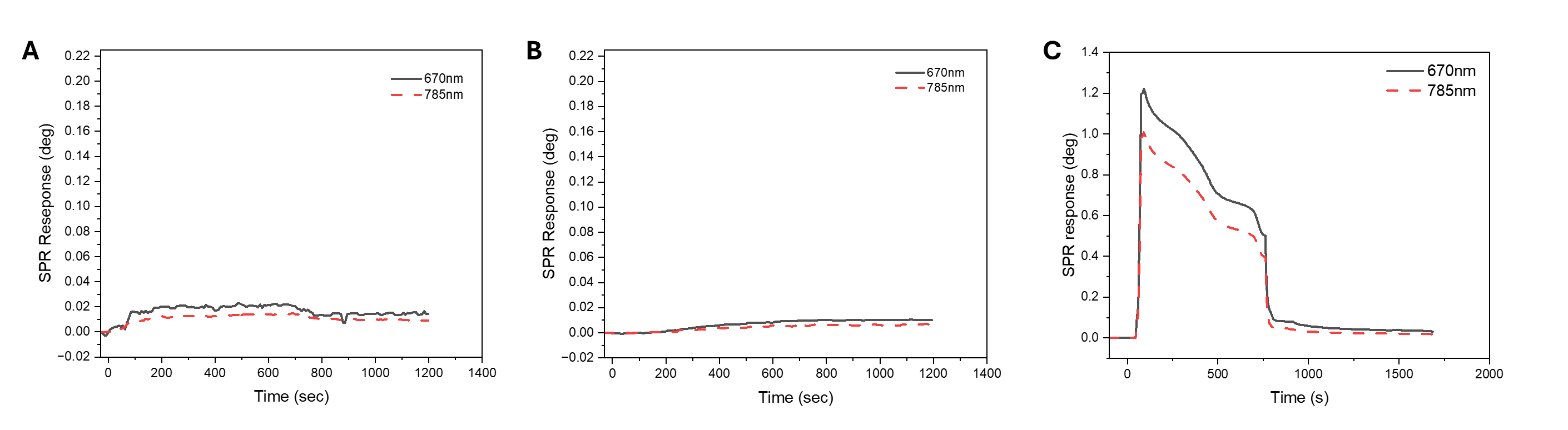
**Figure S11. MP-SPR EV capture and plasma injection controls.** MCF7-EVs (A) and HAS3-EVs (B) injected on avidin-coated SPR sensor without CD9 antibody. (C) Plasma injected on avidin-coated sensor without captured EVs.

**Table S2. Total protein from SPR-captured EV samples after detachment and recovery**

|  | MCF7-EVs | HAS3-EVs | Plasma-exposed MCF7-EVs | Plasma-exposed HAS3-EVs |
| --- | --- | --- | --- | --- |
| Protein (µg) | 4.9 ± 2.1 | 8.8 ± 3.9 | 15.6 ±2.6 | 16.6 ±1.3 |


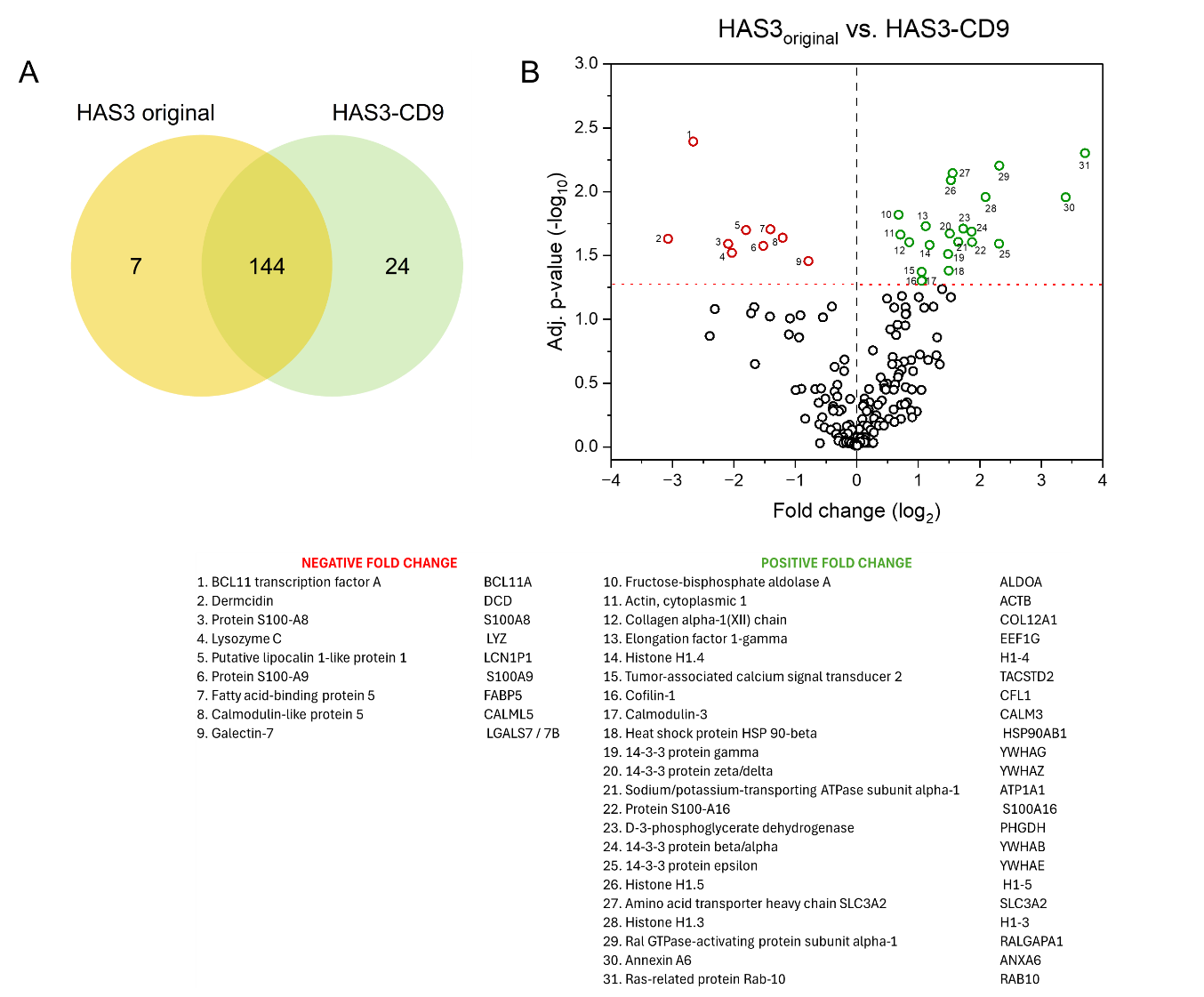


**Figure S12. Comparison of proteomes between non-captured and CD9-captured HAS3-EVs.** (A) Venn diagram of the number of proteins identified in non-captured (HAS3 original) and CD9-captured (HAS3-CD9) EVs. (B) Volcano plot comparing the abundance of identified proteins between the EVs. Proteins with statistically significant changes in abundance are numbered and listed below the plot.


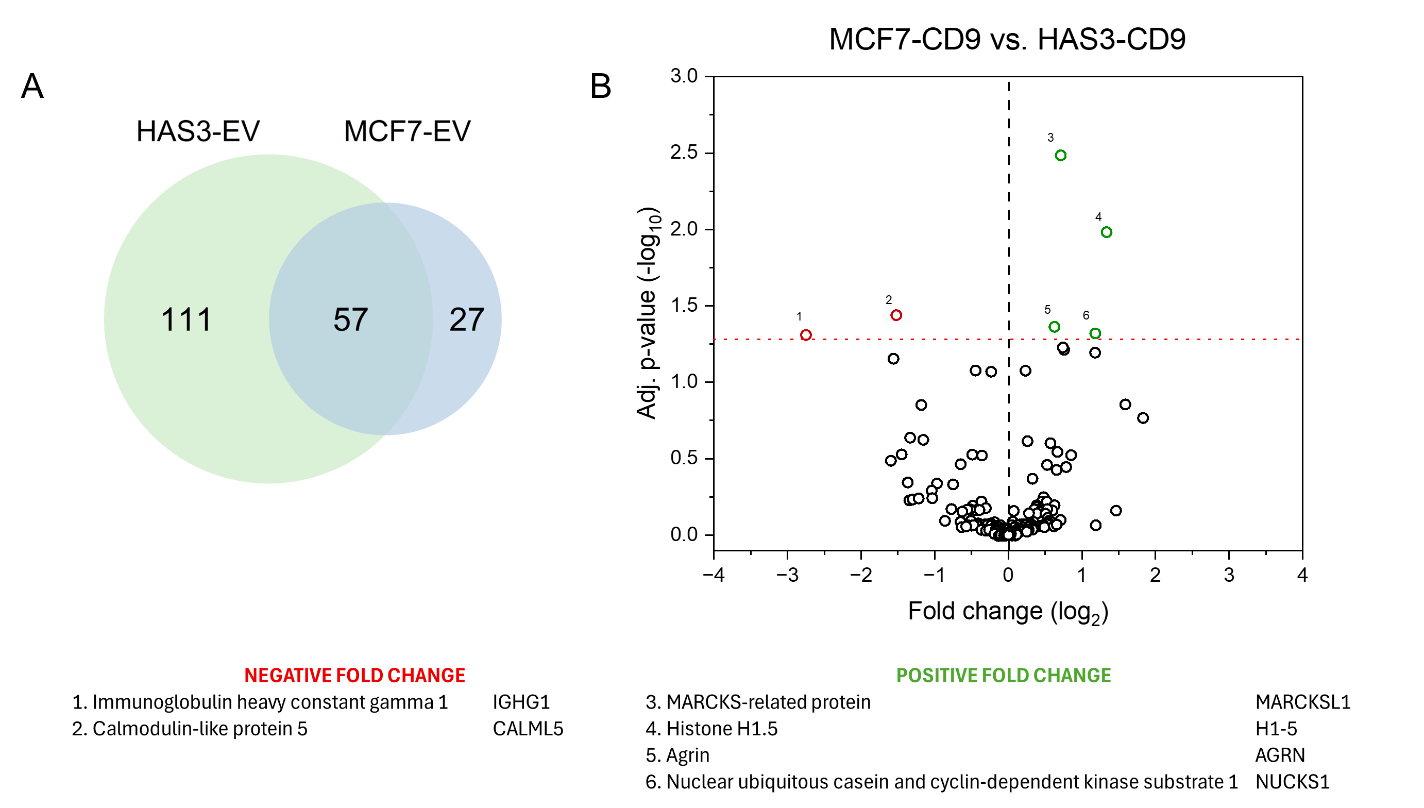


**Figure S13. CD9 captured MCF7-EVs vs. CD9 captured HAS3-EV proteomics.** (A) Venn diagram of the number of proteins identified in CD9-captured HAS3-EVs and MCF7-EVs. (B) Volcano plot comparing the abundance of identified proteins between the EVs. Proteins with statistically significant changes in abundance are numbered and listed below the plot.
